# Enhanced efficacy of tylvalosin tartrate enteric-coated granules against *Mycoplasma hyopneumoniae* in pigs: *in vitro* activity and clinical dosage optimization

**DOI:** 10.3389/fmicb.2026.1809157

**Published:** 2026-06-02

**Authors:** Jiajing Li, Xuzheng Zhou, Weiwei Wang, Hongxing Zhang, Yubin Bai, Bintao Zhai, Fusheng Cheng, Rongbin Hu, Bing Li, Yaxin Zhou, Mingze Cao, Tenghe Ma, Zhen Zhu, Jiyu Zhang

**Affiliations:** 1 Lanzhou Institute of Husbandry and Pharmaceutical Sciences, Chinese Academy of Agricultural Sciences, Lanzhou, China; 2 College of Life Science and Food Engineering, Hebei University of Engineering, Handan, China; 3 Key Laboratory of New Animal Drug Project of Gansu Province, Lanzhou, China; 4 Key Laboratory of Veterinary Pharmaceutical Development, Ministry of Agriculture and Rural Affairs, Lanzhou, China

**Keywords:** clinical efficacy, drug formulation, *in vitro* activity, *Mycoplasma hyopneumoniae*, tylvalosin tartrate

## Abstract

**Background:**

*Mycoplasma hyopneumoniae* (Mhp), the etiological agent of enzootic pneumonia in pigs, poses significant challenges to swine production. This study evaluated the anti-Mhp efficacy of a novel enteric-coated granules of tylvalosin tartrate (ETT) through comprehensive *in vitro* and *in vivo* assessments.

**Methods:**

The *in vitro* evaluation was determined by the minimum inhibitory concentration (MIC) and biofilm inhibition assays. The 60 pigs grouped into six groups were treated with (ETT) at doses of 25, 50, 62.5, 75, or 100 g/1000 kg feed, or with a premix (62.5 g/1000 kg feed) for 7 consecutive days with positive and negative control groups. Efficacy was determined based on clinical symptom scores, pulmonary lesion scores, Mhp antigen, weight gain, and hematological parameters.

**Results:**

*In vitro*, the ETT exhibited significantly enhanced antibacterial activity with a MIC of 0.015625 μg/mL compared to premix (0.125 μg/mL). Additionally, both formulations significantly inhibited the biofilm formation, with enteric-coated granules showed stronger inhibition at 2 MIC. Pre-clinical trial found that nasal swabs collected at 21 dpi showed 100% sIgA detection, while throat swabs at 28 dpi had an 89.47% antigen detection rate. In clinical trial, ETT at 50–75 g/1000 kg feed significant improvements such as lower lung lesion score (*p* < 0.01), 70% cure rate, effective Mhp antigen clearance, and restored weight gain.

**Conclusion:**

The novel ETT showed significantly stronger *in vitro* antibacterial and anti-biofilm activity against Mhp. The clinical trials indicate an optimal dose of 50–75 g per 1,000 kg of feed for 7 days, can improve Mhp infection in pigs.

## 1 Introduction

*Mycoplasma hyopneumoniae* (Mhp) is the primary etiological agent of enzootic pneumonia in swine (Calzas et al., 2021; Huang et al., 2025), damaging the respiratory epithelium and predisposing the pigs to secondary infections (Beuckelaere et al., 2022; Fristiková et al., 2025). Mhp is a fastidious bacterium colonizes the ciliated respiratory tract, leading to chronic disease marked by dry cough, reduced growth, and pulmonary lesions (Almeida et al., 2021a; Sonalio et al., 2024). Disease control relies on management, vaccination, and antimicrobials, however, vaccine efficacy is often limited under field conditions, particularly during co-infections or when optimal vaccination schedules are impractical (Fardisi et al., 2023). Antimicrobial therapy, therefore, remains critical for outbreak management.

Moreover, the *in vivo* antibiotic efficacy not only depends on inherent activity but also on new pharmaceutical formulations, which can influence on stability, bioavailability, and delivery to infection sites. Enteric-coated granules utilize coating technology to prevent the drug from dissolving in stomach juice while allowing it to be released in intestinal fluid, thereby reducing the degradation of the drug by gastric acid and improving its stability. At the same time, this technology can mitigate the bitter taste of the drug, improve palatability and enhance bioavailability. Tylvalosin tartrate premix is known to be effective (Zhang et al., 2023), while novel formulations offer improved antibacterial and anti-virulence activity which remains unclear yet. Additionally, the accurate Mhp detection is essential for evaluating the infection models and treatment success, yet sampling methods including timing and site selection for antigen or antibody (secretory IgA, sIgA) detection are not standardized, thereby affecting the diagnostic sensitivity.

To address following issues, this study was planned to compare the *in vitro* antibacterial and anti-biofilm activities of a novel enteric-coated tylvalosin tartrate granule and a conventional premix against a virulent Mhp strain; and secondly, to evaluate the clinical efficacy and optimize dosage of the enteric-coated granules in experimentally infected pigs. Concurrently, we optimized the Mhp detection protocol by determining the sensitivity at various sampling times and sites for antigen and antibody detection to reliably assess the infection and treatment outcomes.

## 2 Materials and methods

### 2.1 Ethical approval

The study was approved by the Institutional Animal Care and Use Committee of the Lanzhou Institute of Husbandry and Pharmaceutical Sciences (License No. 2025–27).

### 2.2 Inoculum

The lyophilized powder homogenized porcine lung tissue infected with the Js strain was purchased from the Veterinary Research Institute of Jiangsu Academy of Agricultural Sciences (Nanjing, China). The porcine lung tissue infected with the Js strain was obtained from diseased pigs in Jiangsu Province, China.

### 2.3 Animals acclimation and grouping

A total of 100 healthy two-month-old weaned piglets (Duroc × Landrace × Large White crossbred), balanced by sex, were sourced from a commercial breeding farm in Yongdeng County, Gansu Province, China. Animals were maintained in the standardized laboratory animal facility of the Lanzhou Institute of Husbandry and Pharmaceutical Sciences, Chinese Academy of Agricultural Sciences, under controlled conditions (25 ± 3 °C) with ad-libitum access to water. Following a one-week acclimation, pigs were fed a drug-free complete diet at fixed times daily. In this experiment, infection was confirmed in all cases by Mhp antigen and antibody testing. A preliminary trial was conducted on 20 healthy piglets following infection to optimize the sampling protocols (timing, site, and method) for Mhp antigen/antibody detection. This laid the foundation for subsequent trials. The remaining 80 piglets confirmed to be infected with Mhp were used in the main therapeutic trial to evaluate the efficacy of tylvalosin tartrate enteric-coated granules (the formulation was 100 g:20 g (20 million units) of tylvalosin, packaged in 100 g sachets, supplied by a biotechnology company in Shenyang, batch number 24090601) against experimental Mhp infection and to determine the optimum dosage. All piglets were individually ear-tagged and randomly housed in pens (2.4 × 4.5 m, 10 pigs/pen) to minimize individual and environmental variation.

The 80 pigs were randomly divided into eight groups with 10 pigs in each group. The six treatment groups were as grouped as Group A (25 g/1000 kg feed), Group B (50 g/1000 kg feed), Group C (62.5 g/1000 kg feed), Group D (75 g/1000 kg feed), Group E (100 g/1000 kg feed) received of tylvalosin tartrate enteric-coated granules, while Group F (62.5 g/1000 kg feed) received tylvalosin tartrate premix. Group G served as the positive control (infected but untreated), and Group H served as the negative control (healthy). The detail information regarding grouping, dosage, and administration are presented in Table 1.

**Table 1:** Animal grouping and dosage administration.

Group	Drug (calculated as tylvalosin)	Number/group	Method of administration
Group A	tylvalosin tartrate enteric-coated granules 25 g/1000 kg feed	10	Infection, mixed feeding, continuous administration for 7 days
Group B	tylvalosin tartrate enteric-coated granules 50 g/1000 kg feed	10	Infection, mixed feeding, continuous administration for 7 days
Group C	tylvalosin tartrate enteric-coated granules 62.5 g/1000 kg feed	10	Infection, mixed feeding, continuous administration for 7 days
Group D	tylvalosin tartrate enteric-coated granules 75 g/1000 kg feed	10	Infection, mixed feeding, continuous administration for 7 days
Group E	tylvalosin tartrate enteric-coated granules 100 g/1000 kg feed	10	Infection, mixed feeding, continuous administration for 7 days
Group F	tylvalosin tartrate premix 62.5 g/1000 kg feed	10	Infection, mixed feeding, continuous administration for 7 days
Group G	–	10	Infection, no medication
Group H	–	10	No infection, no medication

### 2.4 *In vivo* efficacy and dosage optimization

The optimal dosage of tylvalosin tartrate enteric-coated granules was determined by treating the artificially infected pigs with the frozen lung tissue homogenate according to the specific trial arrangements presented in Table 2.

**Table 2:** Experimental design from 0 dpi to 49 dpi.

Trial type	Days	Operational details
Preliminary trial	0’dpi	Healthy pigs (*n* = 20) were artificially infected with the Js strain.
	7’ dpi – 28’ dpi	Respiratory samples were collected weekly and sent to the laboratory for Mhp antigen and antibody analysis.
Formal trial	0 dpi	Healthy pigs (*n* = 80) were artificially infected with the Js strain.
	28 dpi	Based on preliminary trial results, respiratory samples were collected for detection of Mhp antigen and antibodies. All trial pigs underwent clinical examination and weighing, with respiratory and blood samples collected for laboratory analysis. Subsequently, all groups except the infected control and healthy groups received the drug treatment.
	35 dpi	Discontinue administration and continue observation for 14 days.
	49 dpi	Upon conclusion of the trial, all pigs not withdrawn underwent clinical examination and weighing. Respiratory tract samples and blood samples were collected for laboratory analysis. Three pigs were randomly selected from each trial group for post-mortem examination to observe lung tissue condition. All pigs were euthanised by cardiac extermination following the electrical stunning, and their carcasses are disposed off in accordance with biosecurity protocols.

Pre-trial protocol: After 1 week of acclimatization, piglets (*n* = 20) were experimentally inoculated with 5 mL the frozen lung tissue homogenate. Respiratory samples were collected at 7, 14, 21, and 28 days post-infection (dpi) for antigen and antibody detection. The samples were collected from upper respiratory tract (nasal, oropharyngeal, and laryngeal swabs) and lower respiratory tract (bronchial swabs, bronchoalveolar lavage fluid, and lung tissue).

Formal trial protocol: This was a parallel, randomized, blind, and controlled study. Following the acclimatization, an Mhp infection model was established in weaned piglets (n = 70) by administering 5 mL of the frozen lung tissue homogenate via tracheal injection, with infection confirmed by antigen and antibody detection. Pigs were then treated according to their assigned treatment dosage. At 49 dpi, clinical examination, scoring, weighing, temperature measurement, sample collection (throat swabs, blood), and necropsy were performed on live pigs. Efficacy and dosage was assessed based on cure rate, mortality, Mhp antigen clearance, weight gain, hematological parameters, and clinical and lung lesion scores. Following the sample collection, all pigs were humanely euthanized by electrical stunning and exsanguination, with subsequent carcass disposal.

### 2.5 *In vitro* experiments

#### 2.5.1 Preparation of Mhp inoculum and identification

The lyophilized powder was reconstituted in KM2 cell-free medium (purchased from the Jiangsu Academy of Agricultural Sciences, China) followed by incubation at 37 °C until a color change from red to yellow, indicating bacterial growth. The culture was passaged 2–3 times at a 1:10 ratio in KM2 medium, with a 2-day interval between each subculture. The final culture was stored at −80 °C as the original stock for subsequent experiments. The presumptive identification was done by staining with Giemsa stain and examined under a light microscope. The colony observation was done by culturing on KM2 agar and incubated inverted at 37 °C until the medium turned yellow or pinhead-sized colonies appeared and then examined under a stereomicroscope. The final identification was done by PCR referring to prior study (Shao, 2022). Briefly, the PCR amplification targeted a 948 bp fragment of the *p36* gene using the primers p36-F (5’-CCTTAAATATTTTTAATTGCATCCTG-3’) and p36-R (5’-CGCATGAAACCTATTAAAATAGCTC-3’). The PCR was performed using the 25 μL reaction mixture (12.5 μL 2 × Taq Master Mix, 1 μL each primer, 4 μL template DNA, and 6.5 μL ddH₂O) at cycles of 95 °C for 5 min; 30 cycles at 94 °C for 30 s, 55 °C for 30 s, and 72 °C for 60 s followed by final extension at 72 °C for 10 min. Amplification product was run on 1% agarose gel electrophoresis (120 V, 30 min) and visualized using Image system.

#### 2.5.2 Mhp titration assay

Due to the slow growth of Mhp on agar media, the color change unit (CCU) assay was used for quantification. A 10-fold serial dilutions of the bacterial suspension were prepared in KM2 broth in 11 tubes, with the 11th serving as a negative control. After incubation at 37 °C, the titer was recorded as the highest dilution showing a color change. The average of triplicate was recorded as the final CCU/mL.

#### 2.5.3 Determination of minimum inhibitory concentration

The MICs of 15 clinical antibiotics and two tylvalosin tartrate formulations against Mhp Js were determined using the microbroth dilution method (Jaÿ et al., 2025). Antibiotics tested included tetracycline, oxytetracycline, doxycycline, tylosin, tilmicosin, tylvalosin, azithromycin, gentamicin, kanamycin, enrofloxacin, ciprofloxacin, chloramphenicol, florfenicol, tiamulin, and lincomycin. With the exception of tiamulin, tylvalosin and tylosin, which were purchased from MCE (China) Medchemexpress Co.,ltd, all other antibiotics were purchased from Beijing Solarbio Science and Technology Co., Ltd. All antibiotics were prepared as 12.8 mg/mL stock solutions, sterilized using 0.22 μm filters, aliquoted into sterile 2 mL Eppendorf tubes, and stored at 4 °C. Dilute each drug stock solution with KM2 medium to a concentration of 16 μg/mL to serve as the working concentration for subsequent experiments. A bacterial suspension adjusted to 10^4^ CCU/mL was added to wells containing serial two-fold dilutions of each antibiotic (final range: 0.0039–4 μg/mL). Each plate included negative (medium only) and positive (bacteria without drug) controls. All tests were performed in triplicate. Plates were incubated at 37 °C and the MIC was defined as the lowest drug concentration at which no color change observed.

#### 2.5.4 Biofilm formation inhibition assay

The effect of tylvalosin tartrate formulations on Mhp biofilm formation was assessed as previously described (Petrelli et al., 2006). Briefly, Mhp suspension (10^8^ CCU/mL) was incubated with each drug at concentrations of 2 × MIC, MIC, and 1/2 × MIC for 3 days at 37 °C. After incubation, non-adherent cells were removed by washing with phosphate buffered saline (PBS). Biofilms were fixed with methanol, stained with 1% crystal violet, and dissolved in 95% ethanol. Absorbance was measured at 570 nm (OD_570_ nm). All assays included negative and positive controls and were performed in triplicate.

### 2.6 *In vivo* experiments

#### 2.6.1 Establishment of infection model

The Js strain inoculum was prepared by reconstituting lyophilized material in sterile PBS to a final concentration of 1 × 10^9^ CCU/mL. Prior to infection, piglets were screened via serology to exclude pre-existing Mhp infection or vaccination. In the preliminary and formal trials, 20 and 70 piglets were infected, respectively while 10 piglets were kept as negative control. Artificial infection was given by tracheal injection of 5 mL of the frozen lung tissue homogenate to each pig. This method of challenge has been shown to successfully establish an infection mode (Xiong et al., 2014; Ni et al., 2019).

#### 2.6.2 Sample collection and processing

The upper and lower respiratory tract samples, including nasal, oropharyngeal, and laryngeal swabs were collected from live animals as described earlier (Fablet et al., 2011; Clavijo et al., 2021; Ferreira et al., 2023). After post-mortem, bronchial swabs, bronchoalveolar lavage fluid (BALF; 50 mL sterile PBS instilled and recovered), and lung tissue homogenates (1,5 w/v in PBS) were collected. Blood samples were collected from the anterior jugular sinus at 28 and 49 dpi into serum and EDTA tubes for hematological and serological analysis. Swab samples were immersed in 1 mL PBS at 2–8 °C for ≥2 h. After vortex, the eluate was centrifuged (12,000 rpm, 10 min) and the pellet was used for antigen and sIgA antibody detection.

#### 2.6.3 Mhp antigen and antibody detection for confirmation of suspected cases

At 28 dpi, nasal and oropharyngeal swabs collected from pigs showing clinical signs were tested for detection of Mhp-sIgA antibody via a licensed sIgA-ELISA method (the ELISA Kit for detection of sIgA-Antibody against Mhp, purchased from the Jiangsu Academy of Agricultural Sciences) in China [2018-no.4] according to the sIgA ELISA method (Bai et al., 2018). Samples with S/*p* < 0.15 were negative, >0.20 positive, and 0.15–0.20 suspected.

For the Mhp antigen detection, DNA was extracted and qPCR was performed using a commercial kit (fluorescent PCR nucleic acid detection kit for *Mycoplasma pneumoniae* in Pigs, purchased from Zhongke iCare Biotechnology, China) according to the manufacturer’s protocol. Amplification conditions were 95 °C for 3 min; 45 cycles of 95 °C for 10 s and 60 °C for 30 s (fluorescence collection in FAM channel). A sample with Cycle threshold value (Ct) ≤ 40 and a typical amplification curve was considered positive.

#### 2.6.4 Necropsy and histopathology

At 49 dpi, three pigs were randomly selected from each group. Following electro-stunning, pigs were bled to death via cardiac puncture. Dissection was performed to observe and record pathological changes in different organ such as heart, liver, spleen, lungs, and kidneys. Tissue samples were fixed in 4% paraformaldehyde in 0.01 M PBS, followed by routine processing and paraffin embedding. The films were subsequently stained with hematoxylin and eosin (H&E) and scanned using a digital pathology slide scanner (Servicebio, LG-S80) and its visualization platform (Servicebio, Saiviewer-1.0.9) (Beuckelaere et al., 2025).

#### 2.6.5 Hematological and biochemical analyses

Blood samples were collected from all pigs at 28 and 49 dpi via the anterior vena cava. Transport the anticoagulated blood and the separated serum to the laboratory at a low temperature (2–8 °C) for hematological and biochemical analysis, using hematology analyzer (ProCyte Dx, United States) and automatic biochemical analyzer (Erba XL-640, Germany) respectively. Hematological parameters such as total white blood cell (WBC) and differential counts (neutrophils, lymphocytes, monocytes, eosinophils, basophils), red blood cell (RBC), hemoglobin (HGB), hematocrit (HCT), and platelet (PLT) counts were measured. Biochemical parameters included were alanine aminotransferase (ALT), aspartate aminotransferase (AST), total bilirubin (TBIL), direct bilirubin (DBIL), total protein (TP), albumin (ALB), alkaline phosphatase (ALP), *γ*-glutamyl transferase (GGT), creatinine (CRE), triglycerides (TG), total cholesterol (TCH), lactate dehydrogenase (LDH), creatine kinase (CK), glucose (GLU), and calcium (Ca).

#### 2.6.6 Therapeutic efficacy evaluation

Therapeutic efficacy of drug was assessed at 49 dpi. Key evaluation metrics included were cure rate, effective rate, mortality, Mhp antigen clearance, weight gain, hematological and biochemical parameters, as well as lung lesion and clinical symptom scores. The specific assignments of clinical symptom score and lung tissue score were as follows:

##### 2.6.6.1 Clinical symptom score

Each test pig was scored and scores were calculated for each group according to the clinical examination scoring criteria. The clinical symptom score is equal to the sum of body temperature, respiration and status score, i.e.: clinical symptom score = body temperature score + respiration score + status score.

① Body temperature score (0–3 points) as listed in Table 3:
② Respiratory scores (0 to 3), as shown in Table 4:
③ Status scores (0 to 3) are shown in Table 5:

**Table 3:** Body temperature scoring.

Body temperature	Score
Severe (anal temperature > > 41.0°C)	3 points
Moderate (anal temperature 40.6–41.0°C)	2 points
Mild (anal temperature 39.6–40.5°C)	1 point
Normal (anal temperature 38.5–39.5 °C)	0 point

**Table 4:** Respiratory scoring.

Respiratory	Score
Severe (abdominal breathing, wheezing, violent coughing)	3 points
Moderate (markedly increased respiratory rate, and/or persistent cough)	2 points
Mild (slight increase in respiratory rate, and/or occasional cough)	1 point
Normal (steady breathing, no sound, no cough)	0 point

**Table 5:** State scoring.

Status	Score
Loss of appetite and prolonged prostration	3 points
Significantly reduced appetite, more lying down and less standing up	2 points
Normal appetite, slightly reduced activity	1 point
Normal appetite, normal activity and responsiveness	0 point

##### 2.6.6.2 Lung lesion score

Porcine lung lesions were scored according to the 28-point scale counting method (Garcia-Morante et al., 2016). The percentage of specific lesion area in each lung was recorded for the left apical lobe, left heart lobe, left diaphragm lobe, right apical lobe, right heart lobe, right diaphragm lobe, and parietal lobes, as shown in Table 6.

**Table 6:** Scoring criteria for pulmonary lesions in each lobe.

Percentage of specific lesion area (%)	Score
>75%	4 points
51 ~ 75%	3 points
26 ~ 50%	2 points
1 ~ 25%	1 point
0	0 point

The scores assessed for each lung lobe were then summed up to be the lesion scoring value for the whole lung (regardless of the size of each lobe), up to a maximum of 28 points. Specific method of the scores were observed on the ventral surface of the parietal lobes of the lungs and the ventral and dorsal surfaces of the other lobes of the lungs of the test pigs, respectively. The scores obtained were then used to calculate the total score for the whole lung using the following formula: (sum of the dorsal observation scores for each lobe + sum of the ventral observation scores for each lobe)/ 2 + parietal lobe observation score. The final result was rounded to the nearest whole number.

According to the treatment results, the efficacy was classified into four categories: cure, effective, ineffective, and death, and the cure rate, effective rate, ineffective rate, and mortality rate of the animals in each group were calculated, which were used as the main indexes for evaluating the efficacy of the tested drugs. The cure rate, efficacy rate, and ineffective rate were assessed at 49 dpi. Mortality rate was calculated by counting the number of pigs that died in each group during the period from post-infection to 49 dpi, as detailed in Table 7:

**Table 7:** Therapeutic efficacy evaluation criteria.

Therapeutic efficacy indicators	Clinical symptom score	Mhp antigen test result	Lung lesion score	Formula calculation
Cure rate	0 point	N	**	Cure rate (%) = (number of animals with symptom disappearance/number of animals with disease in the group) × 100%
Effective rate	0 ~ 2 points	N	*	Effective rate (%) = (number of animals with symptomatic improvement/number of animals with morbidity in the group) × 100%
Inefficiency rate	3 ~ 9 points	P	NSD	Inefficiency rate (%) = (number of animals with obvious clinical symptoms after drug administration/number of test animals in the group) × 100%
Mortality rate				Mortality rate (%) = (number of pigs that died during the period from post-infection to 49 dpi/number of pigs that received treatment) × 100%

*N* denotes negative, *p* denotes positive, **indicates extremely significant difference compared with the positive control group (*p* < 0.01), *indicates significant difference compared with the positive control group (*p* < 0.05), NSD denotes no significant difference compared with the positive control group (*p* > 0.05).

##### 2.6.6.3 Weight gain

Body weight was measured for all pigs at 28 and 49 dpi. Weight gain was calculated for each pig as the difference between the final (49 dpi) and initial (28 dpi) body weights. The average weight gain was then determined for each experimental group.

### 2.7 Data analysis

The appropriate statistical test was applied in SPSS software v27.0. The clinical symptom scores, lung lesion scores, weight gain, blood hematology and biochemistry parameters were expressed as mean ± standard deviation (Mean ± SD) using the t-test. The cure rates, effective rates, ineffective rates, mortality rates, and the occurrence of adverse reactions of experimental pigs in each group were expressed as percentages (%), and evaluated using the chi-square test (χ2). All statistical tests were carried out at significance level of 0.05. The antibody detection results were calculated as: Infection rate = (Number of positive samples/Total number of samples tested) × 100%; Suspected infection rate = (Number of suspected samples/Total number of samples tested) × 100%.

## 3 Results

### 3.1 Mph identification

**Figure 1:**
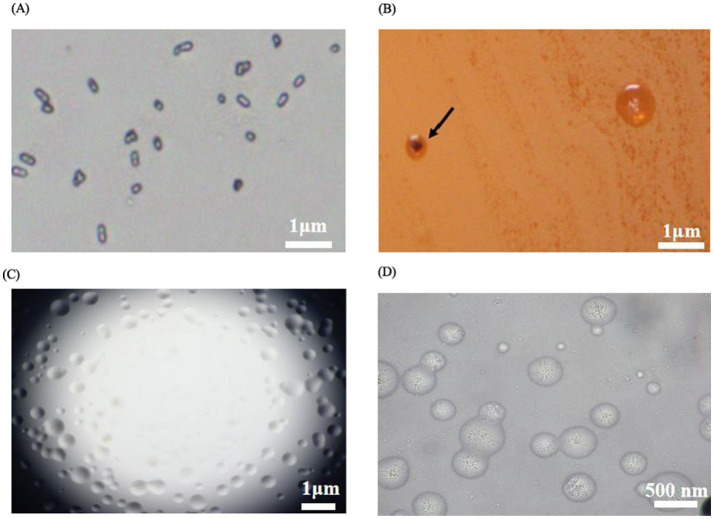
*Mhp* cultivation **(A)** Giemsa staining (1000×); **(B–D)** Microscopy of *Mhp* colonies on agar media.

**Figure 2:**
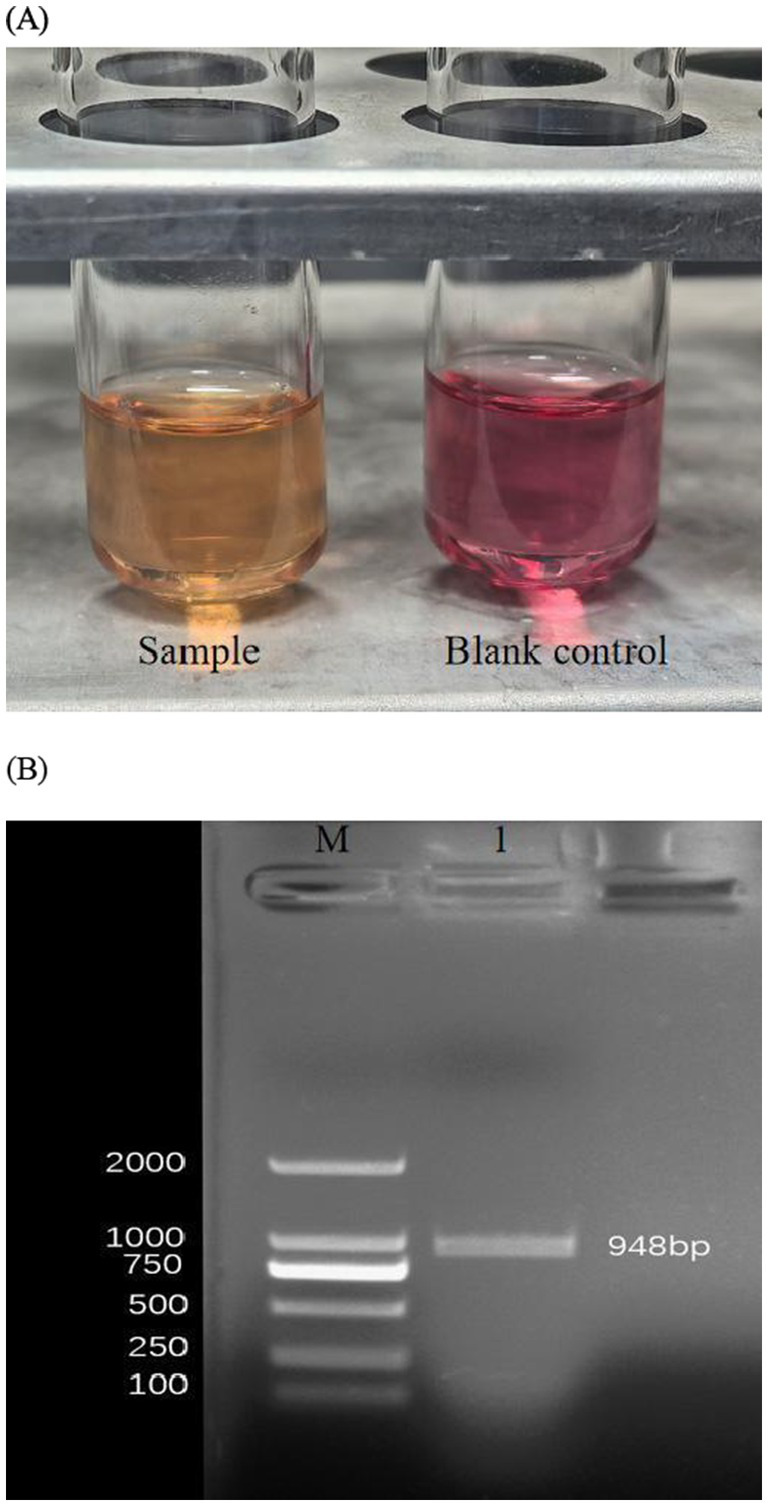
*Mhp* identification **(A)** Color change from red to yellow; **(B)** PCR identification of *p36* gene (948 bp).

The Giemsa staining revealed faintly stained pleomorphic bacterial cells (Figure 1A) while on KM2 agar, characteristic “fried-egg” colonies were observed under a stereomicroscope, demonstrating the typical round morphology of Mhp (Figures 1B–D). The change of broth color from red to yellow indicated bacterial growth (Figure 2A) and PCR confirmed the gel band at 948 bp of p36 gene (Figure 2B).

### 3.2 Results of Mhp titer assay

Mhp cultures started to change color when incubated up to 36 h. After continuous observation, it was found that when Mhp was grown in KM2 medium up to 9 days, its titer could reach a maximum of 1 × 10^9^ CCU/mL, and the results of the incubation are shown in Appendix 1.

### 3.3 MIC determination

**Figure 3:**
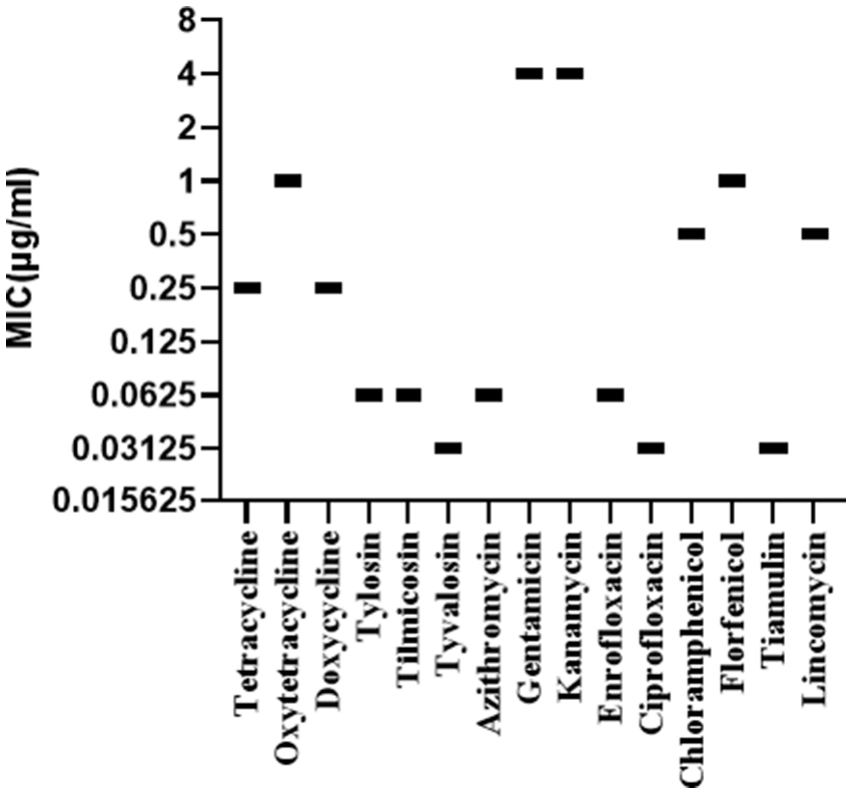
MIC of commonly used drugs against the Js strain.

The MIC of 15 clinical antibiotics against Mhp showed that macrolides (tylosin, tilmicosin, tylvalosin, azithromycin), quinolones (enrofloxacin, ciprofloxacin), pleuromutilins (tiamulin) exhibited stronger inhibitory activity against the Js strain with a MIC of 0.03125 μg/mL or 0.0625 μg/mL (Figure 3). Among the two dosage forms of tyvalosin tartrate, tyvalosin tartrate enteric granules (MIC of 0.015625 μg/mL) showed better inhibition of Mhp compared to tyvalosin tartrate premix (MIC of 0.125 μg/mL) as shown in Appendix 2.

### 3.4 Effect of tylvalosin tartrate on Mhp biofilm formation

**Figure 4:**
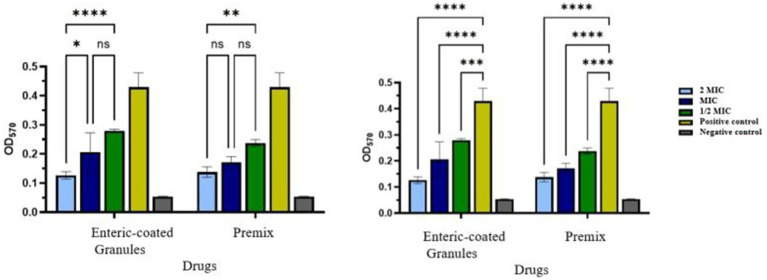
Inhibitory effects of tylvalosin tartrate formulations on biofilm formation of the Js strain. “ns” denotes no significant difference (*p* > 0.05), “*” indicates a significant difference (*p* < 0.05), “**” denotes a significant difference (*p* < 0.01), *** indicates a significant difference (*p* < 0.001), “****” denotes a significant difference (*p* < 0.0001).

The biofilm assay revealed that the Js strain possesses the capacity to form biofilms. Furthermore, within the tylvalosin tartrate enteric-coated granule group, significant differences were observed between 2 MIC dose and both MIC and 1/2 MIC doses (*p* < 0.05), whereas no difference was detected between the MIC and 1/2 MIC doses (*p* > 0.05). In the premix group, no difference was observed between MIC and both 2 MIC and 1/2 MIC doses (*p* > 0.05), but significant differences existed between each 2 MIC dose and the 1/2 MIC dose (*p* < 0.05). All drug dose groups exhibited significant differences compared to the positive control group (*p* < 0.05). Enteric-coated granules showed a dose-dependent inhibition of biofilm formation, with the 2 MIC dose significantly more effective than MIC and 1/2 MIC doses (*p* < 0.05). In contrast, the premix showed significant differences (*p* < 0.05) only between 2 MIC and 1/2 MIC doses (Figure 4).

### 3.5 Preliminary antibody and antigen detection

**Figure 5:**
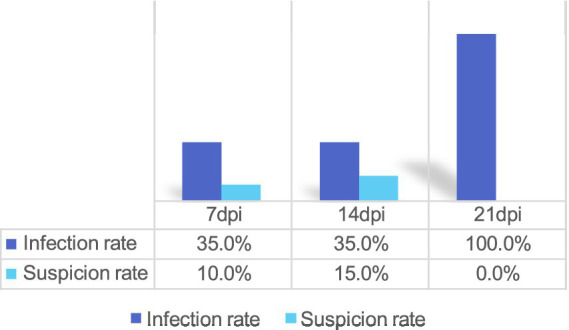
Infection and suspection rate based on antibody detection.

At 7 dpi, Mhp sIgA antibodies were detected in nasal swabs of pigs. At 21 dpi, all sIgA antibody test results of this group were found positive (Figure 5).

The antigen detection in upper respiratory tract samples were done at 7, 14, and 21 dpi showed no detectable Mhp antigen by either PCR or qPCR. However, one pig were died at 26 dpi. Subsequently, upper respiratory tract samples were collected from all pigs (n = 19) at 28 dpi, and five pigs were necropsy to obtain lower respiratory tract samples for testing. The qPCR results (Appendix 3 and 4) demonstrated greater sensitivity than conventional PCR (Appendix 5). Specifically, on 28 dpi, qPCR yielded detection rates of 52.63, 89.47, 63.16, 40.00, and 40.00% for nasal swabs, oropharyngeal swabs, laryngeal swabs, bronchial swabs, bronchoalveolar lavage fluid, and lung tissue, respectively (Table 8).

**Table 8:** Detection results of Mhp antigen in different respiratory samples.

Methods	Variety types	Number of samples	Positive count	Antigen positivity rate
PCR method	Nasal swabs	19	1	5.26%
	Oropharyngeal swabs	19	4	21.05%
	Laryngeal swabs	19	1	5.26%
	Bronchial swabs	5	0	0.00%
	Bronchoalveolar lavage fluid	5	0	0.00%
	Lung tissue milling liquid	5	1	20.00%
qPCR method	Nasal swabs	19	10	52.63%
	Oropharyngeal swabs	19	17	89.47%
	Laryngeal swabs	19	12	63.16%
	Bronchial swabs	5	2	40.00%
	Bronchoalveolar lavage fluid	5	2	40.00%
	Lung tissue milling liquid	5	3	60.00%

### 3.6 Establishment of an artificial infection model

Beginning on 5 dpi, individual pigs in each infection group exhibited body temperatures exceeding 39.5 °C. By 12 dpi, all infected pigs displayed temperatures above 39.5 °C, with some exhibiting a ‘dog-sitting’ posture (Appendix 6) and increased respiratory rate, though without signs of anorexia or prolonged recumbency. In contrast, piglets in the negative control group remained unaffected, showing no adverse symptoms.

On 28 dpi, pharyngeal swabs (n = 80) were collected for Mhp antigen detection. Results indicated that, excluding the negative control group, Mhp antigen was detected in all 70 pigs across the infected groups (Appendix 7).

Similarly, on 28 dpi, nasal swabs from all pigs in eight groups were collected for detection of Mhp antibody. Results indicated that, excluding the negative control group, Mhp antibody was detected in all infected groups (Appendix 8).

### 3.7 Necrospy findings

**Figure 6:**
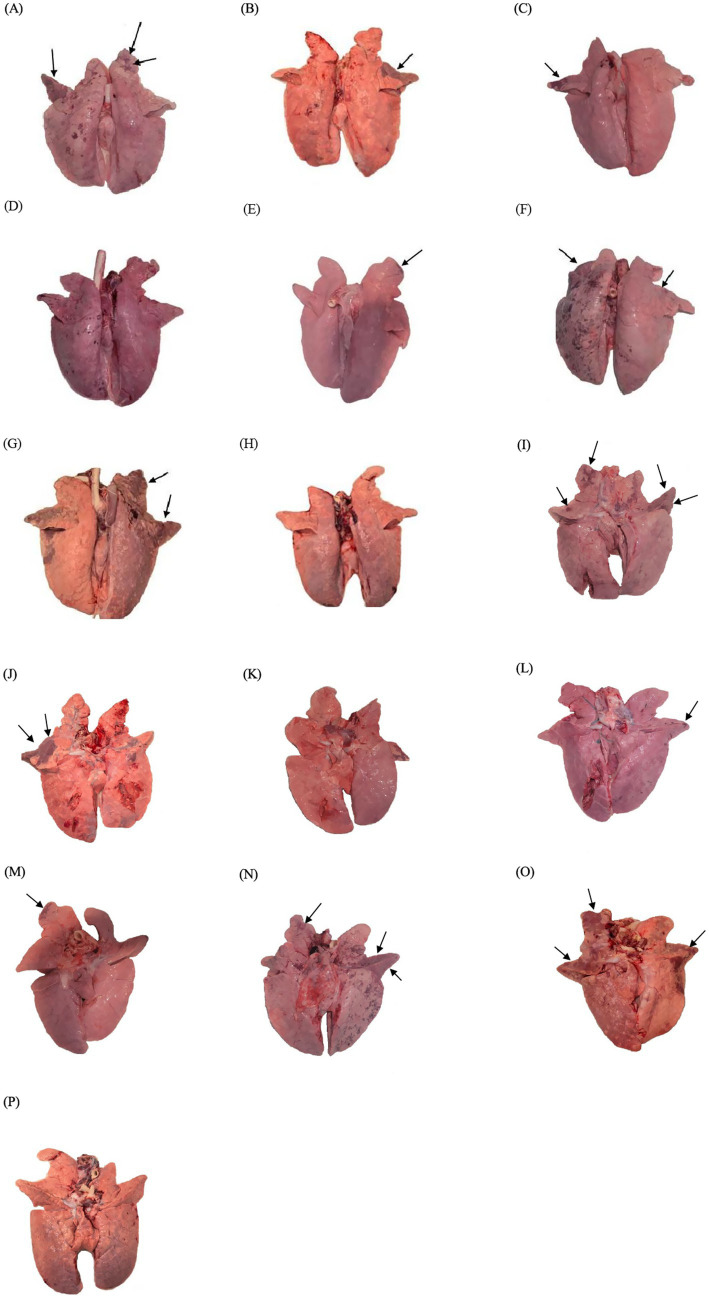
Pathological findings of lung at necropsy from each experimental group. **(A–H)** Correspond to the ventral surfaces of the lungs in groups **A–H**, while **(I–P)** correspond to the dorsal surfaces of the lungs in groups **A–H**.

On 49 dpi, three pigs were randomly selected from each experimental group for necropsy. Necropsy revealed that the lungs of all experimental groups except negative control exhibited varying degrees of ‘meat-like’ lesions. The affected areas presented distinct borders from healthy tissue, appearing as irregular purplish-red ‘meat-like’ lesions. These lesions lacked elasticity, with indentations persisting after digital pressure. Incision of the affected areas revealed muscle-like striations (Figures 6A–G and I–O). The lungs in the negative control group exhibited moderate volume, uniform pale pink colouration, and a moist, lustrous surface. Palpation indicated a soft, elastic texture, with depressions caused by finger pressure immediately rebounding. The margins of the pulmonary lobes were sharply defined, particularly those of the cardiac, apical, and diaphragmatic lobes. Lobes separated readily without fibrous adhesions (Figure 6H and P).

However, positive control group G (Figure 6G and O) exhibited the most extensive pulmonary lesions, with varying degrees of fleshy changes observed in the apical, cardiac, and diaphragmatic lobes. Treatment groups B, C, D, and E showed marked improvement in pulmonary lesions with no significant difference was observed between group E (100 g/1000 kg feed) and D (75 g/1000 kg feed) (*p* > 0.05). No significant lesions were detected in the heart, liver, spleen, and kidneys of any infected group.

### 3.8 Histopathological findings

**Figure 7:**
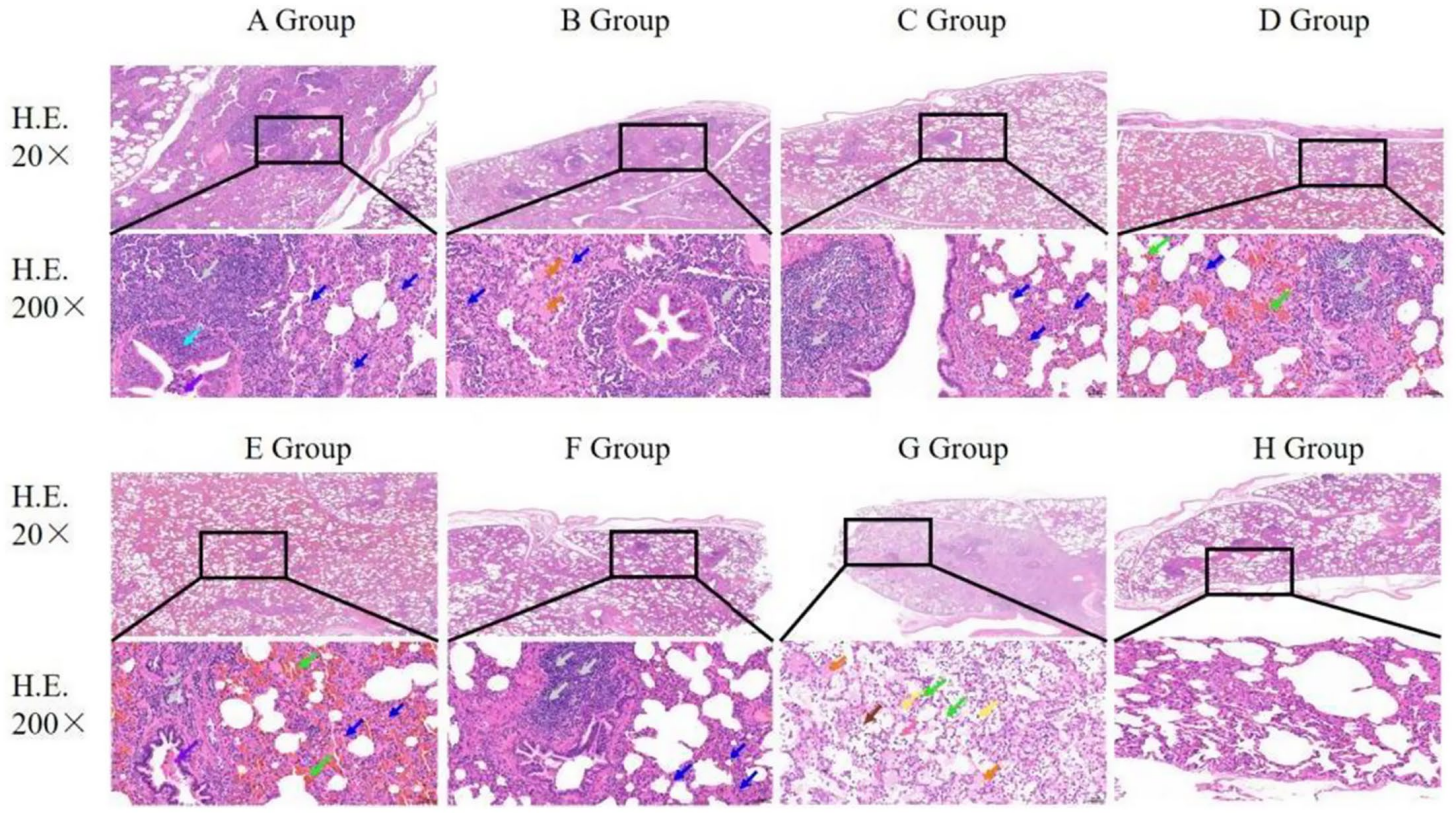
Histopathological sections of lung from each experimental group (H&E staining). Silver arrows denote the focal lymphocytic infiltration; cyan arrows denote lymphocytic infiltration; blue arrows denote granulocytic infiltration; green arrows denote lymphocytic infiltration; orange arrows denote eosinophilic serous exudate; brown arrows indicate leukocytosis; pink arrows signify epithelial cell hyperplasia; purple arrows represent eosinophils, leukocytes, and necrotic cell debris; yellow arrows indicate the macrophage infiltration.

Mhp infection resulted in parenchymal changes within the lung tissue, with rare focal lymphocytic infiltrates observed around bronchioles and blood vessels. Eosinophils, leukocytes, and necrotic cell debris were visible within bronchiolar lumens, while lymphocytic infiltration was noted in the mucosal layer of bronchioles. Alveolar walls show considerable granulocyte infiltration, with numerous alveolar spaces exhibiting lymphocytic and eosinophilic serous exudation. Macrophages are abundantly present within alveolar spaces. Vascular spaces contain numerous leukocytes and minor bronchial epithelial cell hyperplasia (Figure 7).

### 3.9 Hematological and biochemical parameters

At 28 dpi, the total white blood cell count (WBC) in all infected groups exhibited extremely significant differences compared to the negative control group (*p* < 0.01). At 49 dpi, the total white blood cell count (WBC) in all treatment groups decreased but remained significantly different from the negative control group (*p* < 0.05). For all other blood count parameters, no significant differences were observed between the infected groups and the negative control group at either 28 or 49 dpi (*p* > 0.05; Table 9). Similarly, the biochemical parameters in the blood of all infected groups at both 28 and 49 dpi showed no significant differences compared with the negative control group (*p* > 0.05; Table 10).

**Table 9:** Blood count test results.

Goup	Time	Indicator														
		WBC	NEUT	NEUT%	LYMPH	LYMPH%	MONO	MONO%	EO	EO%	BASO	BASO%	RBC	HGB	HCT	PLT
		(K/uL)	(K/uL)	(%)	(K/uL)	(%)	(K/uL)	(%)	(K/uL)	(%)	(K/uL)	(%)	(M/uL)	(g/dL)	(%)	(K/uL)
Goup A	28dpi	21.64 ± 2.85*	4.41 ± 1.36	22.82 ± 3.79	8.67 ± 2.78	55.75 ± 8.65	0.60±0.39	4.39 ± 1.44	0.39 ± 0.22	2.76 ± 0.73	0.00 ± 0.01	0.05 ± 0.05	8.09 ± 2.15	11.21 ± 1.91	40.43 ± 4.26	433.70 ± 97.51
	49dpi	17.00 ± 1.59	4.27 ± 1.65	21.84 ± 3.34	10.31 ± 3.66	57.50 ± 6.50	0.59±0.21	4.32 ± 2.40	0.37 ± 0.19	2.56 ± 1.00	0.01 ± 0.00	0.04 ± 0.04	8.24 ± 2.87	11.03 ± 3.27	41.91 ± 7.60	445.50 ± 122.15
Goup B	28dpi	22.64 ± 2.65*	4.64 ± 0.99	23.03 ± 3.66	8.66 ± 2.07	56.31 ± 7.42	0.61±0.23	4.50 ± 0.87	0.35 ± 0.20	2.80 ± 0.66	0.01 ± 0.01	0.04 ± 0.05	8.38 ± 1.70	11.45 ± 1.04	40.20 ± 3.37	432.50 ± 70.43
	49dpi	17.30 ± 1.99	4.14 ± 1.45	22.20 ± 1.53	10.90 ± 3.62	58.50 ± 4.46	0.58±0.14	4.59 ± 2.01	0.39 ± 0.13	2.65 ± 0.72	0.01 ± 0.02	0.04 ± 0.06	8.88 ± 2.97	11.38 ± 2.93	41.90 ± 7.16	430.50 ± 108.23
Goup C	28dpi	22.08 ± 2.11*	4.54 ± 0.59	23.43 ± 4.22	9.06 ± 1.72	58.10 ± 6.26	0.61±0.07	4.60 ± 0.87	0.35 ± 0.14	3.05 ± 1.07	0.01 ± 0.03	0.04 ± 0.07	8.67 ± 1.72	11.45 ± 0.30	40.50 ± 2.31	452.50 ± 58.73
	49dpi	17.20 ± 2.42	4.54 ± 0.89	21.58 ± 1.59	10.32 ± 2.65	57.60 ± 3.70	0.58±0.12	4.65 ± 1.69	0.40 ± 0.10	2.75 ± 0.65	0.01 ± 0.04	0.04 ± 0.08	8.68 ± 2.26	11.15 ± 2.97	41.95 ± 8.05	444.50 ± 91.77
Goup D	28dpi	21.44 ± 2.11*	4.34 ± 0.41	23.05 ± 3.53	9.02 ± 1.47	57.76 ± 5.43	0.59±0.07	4.20 ± 0.97	0.35 ± 0.09	2.61 ± 0.85	0.01 ± 0.05	0.05 ± 0.05	8.78 ± 1.60	11.45 ± 1.15	40.80 ± 1.40	452.50 ± 35.03
	49dpi	18.50 ± 1.81	4.44 ± 0.80	21.80 ± 2.80	10.96 ± 2.51	58.20 ± 2.24	0.62±0.09	4.83 ± 1.38	0.37 ± 0.11	2.65 ± 0.70	0.01 ± 0.06	0.05 ± 0.05	8.08 ± 1.59	11.15 ± 2.53	41.30 ± 5.59	443.20 ± 66.56
Goup E	28dpi	21.84 ± 1.26*	4.48 ± 0.29	22.45 ± 2.93	8.91 ± 1.05	57.11 ± 4.74	0.59±0.04	4.50 ± 1.29	0.35 ± 0.04	2.70 ± 0.68	0.01 ± 0.07	0.04 ± 0.05	8.58 ± 1.17	11.40 ± 2.10	40.09 ± 1.86	442.50 ± 20.39
	49dpi	18.60 ± 2.07	4.54 ± 0.97	21.50 ± 3.57	11.11 ± 2.06	57.50 ± 1.67	0.62±0.20	4.70 ± 1.06	0.35 ± 0.13	2.55 ± 1.16	0.01 ± 0.08	0.04 ± 0.05	8.48 ± 1.64	11.55 ± 1.92	42.35 ± 4.07	450.10 ± 43.41
Goup F	28dpi	21.06 ± 1.20*	4.57 ± 0.65	23.05 ± 2.48	8.96 ± 0.27	55.91 ± 6.39	0.59±0.14	4.52 ± 1.22	0.35 ± 0.03	2.90 ± 0.96	0.01 ± 0.09	0.04 ± 0.05	8.48 ± 1.12	11.25 ± 1.66	40.86 ± 2.46	441.10 ± 8.17
	49dpi	17.75 ± 2.47	3.87 ± 0.41	22.10 ± 1.94	10.99 ± 1.50	58.86 ± 3.11	0.64±0.21	4.65 ± 1.16	0.39 ± 0.20	2.81 ± 1.43	0.01 ± 0.10	0.04 ± 0.05	8.64 ± 1.16	11.10 ± 1.19	41.00 ± 6.61	446.10 ± 18.41
Goup G	28dpi	21.64 ± 0.83**	4.60 ± 1.02	22.89 ± 1.72	9.14 ± 0.81	56.51 ± 2.67	0.59±0.24	4.38 ± 0.94	0.36 ± 0.08	3.10 ± 1.39	0.01 ± 0.11	0.04 ± 0.05	8.58 ± 0.84	11.25 ± 0.65	41.16 ± 3.39	447.50 ± 9.68
	49dpi	20.11 ± 1.48**	3.83 ± 0.49	21.60 ± 1.53	10.62 ± 1.14	58.25 ± 2.65	0.61±0.11	4.77 ± 1.13	0.37 ± 0.11	2.97 ± 1.37	0.01 ± 0.12	0.04 ± 0.05	8.98 ± 1.58	11.15 ± 1.17	42.45 ± 7.16	438.50 ± 31.63
Goup H	28dpi	13.58 ± 2.63	4.40 ± 1.39	22.37 ± 2.20	9.44 ± 1.30	57.81 ± 3.42	0.57±0.33	4.52 ± 0.80	0.36 ± 0.13	3.05 ± 1.36	0.01 ± 0.13	0.04 ± 0.05	8.28 ± 0.69	11.37 ± 0.74	40.76 ± 2.72	469.50 ± 17.90
	49dpi	13.39 ± 1.29	4.01 ± 0.87	22.60 ± 2.56	10.64 ± 0.97	56.99 ± 3.41	0.62±0.09	4.86 ± 1.24	0.38 ± 0.09	2.95 ± 1.08	0.01 ± 0.14	0.04 ± 0.05	8.88 ± 0.96	11.25 ± 0.75	42.00 ± 7.75	433.00 ± 38.82

**indicates a highly significant difference compared with Group H (*p* < 0.01); *indicates a significant difference compared with Group H (*p* < 0.05); no symbol indicates no significant difference compared with Group H (*p* > 0.05). This data represents blood count test results before and after administration. The table contains the following parameters for each experimental group at 28 dpi and 49 dpi: WBC, NEUT, NEUT%, LYMPH, LYMPH%, MONO, MONO%, EO, EO%, BASO, BASO%, RBC, HGB, HCT, PLT.

**Table 10:** Blood biochemical test results.

Goup	Time	Indicator														
		ALT	AST	TBIL	DBIL	TP	ALB	ALP	GGT	CR	TG	TCH	LDH	CK	GLU	Ca
		(U/L)	(U/L)	(μmol/L)	(μmol/L)	(g/L)	(g/L)	(U/L)	(U/L)	(μmol/L)	(mmol/L)	(mmol/L)	(U/L)	(U/L)	(mmol/L)	(mmol/L)
Goup A	28dpi	41.38 ± 4.73	38.04 ± 1.89	0.84 ± 0.03	1.05 ± 0.51	44.67 ± 1.6	20.21 ± 0.41	92.38 ± 3.75	20.00 ± 4.57	93.12 ± 2.27	0.86 ± 0.05	2.22 ± 0.01	472.52 ± 64.91	903.74 ± 220.85	6.46 ± 0.36	2.35 ± 0.30
	49dpi	40.80 ± 5.68	38.24 ± 2.40	0.88 ± 0.02	0.92 ± 0.46	44.51 ± 1.22	20.02 ± 0.28	92.32 ± 9.36	19.99 ± 4.23	92.02 ± 3.37	0.81 ± 0.06	2.13 ± 0.01	481.12 ± 49.40	914.63 ± 294.85	5.96 ± 0.35	2.08 ± 0.50
Goup B	28dpi	41.63 ± 5.55	38.21 ± 2.37	0.86 ± 0.03	1.11 ± 0.27	44.26 ± 1.61	20.46 ± 0.35	92.03 ± 7.73	20.86 ± 5.50	92.91 ± 3.20	0.83 ± 0.07	2.23 ± 0.01	458.81 ± 58.74	963.72 ± 230.41	6.21 ± 0.37	2.27 ± 0.32
	49dpi	40.89 ± 4.28	38.19 ± 1.86	0.91 ± 0.02	0.91 ± 0.35	44.29 ± 1.57	20.16 ± 0.34	92.32 ± 8.07	20.46 ± 5.52	92.45 ± 3.13	0.84 ± 0.07	2.11 ± 0.02	479.53 ± 58.19	896.33 ± 240.83	5.97 ± 0.33	2.10 ± 0.24
Goup C	28dpi	41.11 ± 5.58	38.14 ± 3.52	0.86 ± 0.03	1.18 ± 0.30	44.85 ± 1.55	20.37 ± 0.42	92.01 ± 6.19	20.63 ± 3.38	93.77 ± 3.54	0.81 ± 0.06	2.21 ± 0.02	455.47 ± 47.29	807.19 ± 200.82	6.33 ± 0.27	2.21 ± 0.30
	49dpi	41.13 ± 5.58	38.27 ± 1.68	0.88 ± 0.03	1.09 ± 0.37	43.51 ± 1.61	19.89 ± 0.40	92.85 ± 6.31	20.66 ± 5.20	92.35 ± 2.19	0.82 ± 0.08	2.20 ± 0.12	475.31 ± 46.5	947.87 ± 194.46	6.15 ± 0.3	2.15 ± 0.40
Goup D	28dpi	41.36 ± 4.14	38.24 ± 2.51	0.85 ± 0.04	1.05 ± 0.28	44.74 ± 1.46	20.38 ± 0.37	92.61 ± 7.06	20.94 ± 4.34	93.03 ± 3.70	0.84 ± 0.06	2.23 ± 0.01	473.28 ± 52.97	827.55 ± 260.71	6.33 ± 0.27	2.37 ± 0.21
	49dpi	41.88 ± 3.47	38.67 ± 2.20	0.81 ± 0.02	0.94 ± 0.39	44.29 ± 1.33	19.95 ± 0.34	92.07 ± 9.23	22.27 ± 4.96	93.01 ± 2.07	0.80 ± 0.05	2.12 ± 0.02	477.62 ± 45.56	942.67 ± 274.37	6.05 ± 0.31	2.23 ± 0.32
Goup E	28dpi	41.45 ± 6.20	38.42 ± 3.04	0.81 ± 0.07	1.00 ± 0.16	44.91 ± 1.63	20.52 ± 0.27	92.79 ± 8.27	20.77 ± 2.69	93.23 ± 3.02	0.88 ± 0.06	2.23 ± 0.01	470.73 ± 70.25	883.42 ± 238.15	6.50 ± 0.34	2.34 ± 0.29
	49dpi	41.86 ± 4.39	38.64 ± 2.46	0.86 ± 0.03	0.85 ± 0.16	45.03 ± 1.57	20.33 ± 0.33	92.74 ± 8.33	19.79 ± 4.82	91.48 ± 2.56	0.83 ± 0.07	2.22 ± 0.02	498.09 ± 59.38	889.02 ± 248.59	6.40 ± 0.32	2.39 ± 0.36
Goup F	28dpi	41.33 ± 3.34	38.56 ± 2.17	0.85 ± 0.09	1.13 ± 0.14	44.24 ± 1.29	20.62 ± 0.41	92.61 ± 8.67	20.15 ± 4.73	92.52 ± 2.88	0.87 ± 0.08	2.23 ± 0.02	472.10 ± 67.83	990.8 ± 251.34	6.49 ± 0.37	2.35 ± 0.27
	49dpi	41.25 ± 4.61	38.86 ± 2.77	0.87 ± 0.03	0.87 ± 0.30	44.99 ± 1.84	20.23 ± 0.37	92.70 ± 8.31	18.81 ± 3.81	92.09 ± 2.44	0.76 ± 0.06	2.22 ± 0.02	473.3 ± 61.15	958.71 ± 261.77	6.51 ± 0.34	2.39 ± 0.20
Goup G	28dpi	41.1 ± 6.20	38.33 ± 3.49	0.86 ± 0.08	1.03 ± 0.19	44.42 ± 1.34	20.58 ± 0.31	92.06 ± 9.26	20.85 ± 3.92	93.02 ± 3.78	0.86 ± 0.11	2.22 ± 0.02	458.72 ± 55.85	849.46 ± 283.83	6.54 ± 0.32	2.31 ± 0.38
	49dpi	41.34 ± 5.07	38.64 ± 3.16	0.84 ± 0.03	0.86 ± 0.20	44.97 ± 1.63	20.44 ± 0.45	92.85 ± 6.72	19.25 ± 5.38	91.14 ± 2.54	0.79 ± 0.09	2.22 ± 0.02	481.02 ± 47.61	833.81 ± 314.77	6.28 ± 0.33	2.28 ± 0.28
Goup H	28dpi	41.95 ± 5.11	38.67 ± 3.34	0.86 ± 0.07	1.04 ± 0.22	44.84 ± 1.70	20.39 ± 0.42	92.39 ± 7.02	20.09 ± 4.06	92.79±3.36	0.86 ± 0.10	2.22 ± 0.02	448.41 ± 79.40	945.48 ± 376.78	6.55 ± 0.22	2.27 ± 0.38
	49dpi	41.33 ± 3.80	38.04 ± 2.92	0.84 ± 0.04	0.89 ± 0.20	44.69 ± 1.41	20.23 ± 0.32	92.57 ± 9.35	21.37 ± 5.22	93.36±1.87	0.8 ± 0.08	2.21 ± 0.02	493.45 ± 62.91	951.25 ± 291.33	6.36 ± 0.22	2.25 ± 0.14

**indicates a highly significant difference compared with Group H (*p* < 0.01); *indicates a significant difference compared with Group H (*p* < 0.05); no symbol indicates no significant difference compared with Group H (*p*>0.05). This data represents blood biochemical test results before and after administration. The table contains data for each experimental group at 28 dpi and 49 dpi for the following parameters: ALT, AST, TBIL, DBIL, TP, ALB, ALP, GGT, CR, TG, TCH, LDH, CK, GLU, *Ca.*

### 3.10 Efficacy evaluation

The dose-screening trial employed the following parameters: cure rate, efficacy rate, non-efficacy rate, mortality rate, weight gain, hematological examination, Mhp antigen positivity rate, pulmonary lesion score, and clinical symptom score at 49 dpi. to evaluate the therapeutic efficacy of tylvalosin tartrate enteric-coated granules against artificially infected *Mycoplasma pneumonia* (MPS) and determined optimal dosage regime. The results were compared with control drug group (tylvalosin tartrate premix) using the statistical analysis.

#### 3.10.1 Clinical symptom scoring

**Figure 8:**
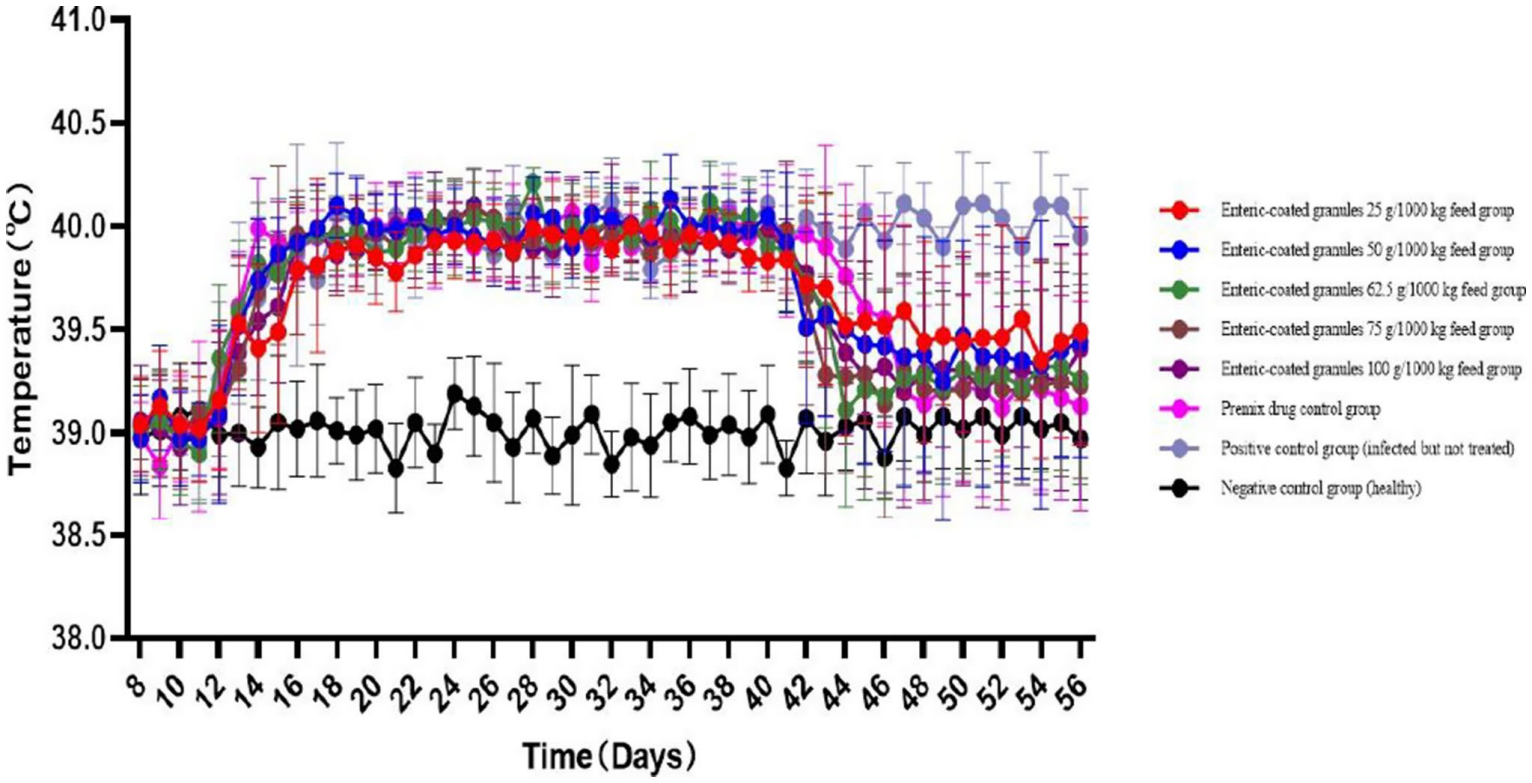
Average body temperature changes across the experimental groups.

All experimental animals underwent systematic assessment at 28 dpi (Appendix 9) and 49 dpi (Appendix 10). During the trial period, the clinical symptoms such as body temperature, respiration rate, and behavioral status of the pigs in each group were recorded. The dynamic variations in the average body temperature of pigs in each group is shown in Figure 8. On day 6 post-administration (i.e., 34 dpi), the body temperatures of pigs in groups B, C, D, E, and F began to return to normal and remained within the normal physiological range (Figure 8). The respiratory symptoms of only isolated pigs in all infected groups exhibited tachypnoea at 28 dpi and 49 dpi; respiratory function remained largely normal in most individuals during other periods. The vast majority of the experimental pigs in the treated groups exhibited normal behavior throughout the entire trial period, including good appetite, free movement, and alert responsiveness. Only on 28 dpi were noted instances of the ‘dog sitting’ posture observed in individual pigs.

#### 3.10.2 Pulmonary lesion score

The 28-point scoring system for porcine pulmonary lesions was employed, with specific pulmonary lesion scores are recorded in Table 11.

**Table 11:** Pulmonary lesion score in each treatment group at 49 dpi.

Group	Apical lobe		Cardial lobe		Diaphragmatic lobe		Accessory lobe	Total score
	Left apical lobe	Right apical lobe	Left cardial lobe	Right cardial lobe	Left diaphragmatic lobe	Right diaphragmatic lobe		
Group A	1	1	4	1	0	0	0	7
	1	1	2	3	0	0	0	7
	2	2	1	2	0	0	0	7
Group B	0	0	1	2	0	0	0	3
	2	1	0	0	0	0	0	3
	1	1	1	1	0	0	0	4
Group C	0	0	1	0	0	0	0	1
	0	1	0	0	0	0	0	1
	0	0	0	0	0	0	0	0
Group D	0	0	0	0	0	0	0	0
	0	0	0	0	0	0	0	0
	0	0	0	1	0	0	0	1
Group E	0	1	0	0	0	0	0	1
	0	0	0	0	0	0	0	0
	0	0	0	0	0	0	0	0
Group F	0	1	3	1	1	0	0	6
	0	1	2	0	0	0	0	3
	1	2	1	0	0	0	0	4
Group G	1	1	1	2	1	1	0	7
	1	1	2	2	1	1	0	8
	1	1	1	2	1	1	0	7
Group H	0	0	0	0	0	0	0	0
	0	0	0	0	0	0	0	0
	0	0	0	0	0	0	0	0

The efficacy of the drug was evaluated based on clinical symptom scores, pulmonary lesion scores, and antigen detection results on 49 dpi (Appendix 11).

As shown in Table 12, the clinical symptom scoring revealed no significant differences (*p* > 0.05) in the overall clinical symptom scores among the experimental pigs before and after administration. Post-treatment (49 dpi) lung lesion scores revealed extremely significant differences between groups A and G compared to Group H (*p* < 0.01). Moreover, Groups B to F showed significant differences compared to Group H (*p* < 0.05); whereas Groups C to E show no significant differences compared with Group H (*p* > 0.05). Throat swab testing of Mhp antigen revealed persistent pathogen carriage in some groups. Specifically, five pigs tested positive in Group A, four in Group B, and two in Groups C, D, E, and F each. Combined analysis of lung lesion scores, antigen positivity rates, cure rates, efficacy rates, non-efficacy rates, and mortality rates further demonstrated that dosages administered in Groups B to E effectively suppressed and eradicated Mhp, thereby treating MPS. From Group B to Group D, therapeutic efficacy increased with escalating dosage, demonstrating a degree of dose dependency. However, further increasing the dosage beyond that of Group D in Group E did not yield additional therapeutic improvement, indicating no marked dose–response effect within this range (Table 12).

**Table 12:** Comparison of therapeutic effects among pig groups before and after administration.

Groups	Time	Clinical symptoms scoring	Pulmonary lesions score	Antigen positive	Cure rate (%)	Efficient (%)	Inefficiency (%)	Mortality rate (%)
Group A	28dpi	1.10 ± 0.70		10	–	–	–	–
	49dpi	0.50 ± 0.50	7.00 ± 0.00**	5	30	50	50	0
Group B	28dpi	1.00 ± 0.45		10	–	–	–	–
	49dpi	0.40 ± 0.49	3.33 ± 0.58*	4	60	60	40	0
Group C	28dpi	1.10 ± 0.54		10	–	–	–	–
	49dpi	0.20 ± 0.40	0.67 ± 0.58	2	70	80	20	0
Group D	28dpi	1.10 ± 0.54		10	–	–	–	–
	49dpi	0.20 ± 0.42	0.33 ± 0.58	2	70	80	20	0
Group E	28dpi	0.90 ± 0.54		10	–	–	–	–
	49dpi	0.30 ± 0.46	0.33 ± 0.58	2	70	80	20	0
Group F	28dpi	0.90 ± 0.54		10	–	–	–	–
	49dpi	0.20 ± 0.40	4.33 ± 1.53*	2	70	80	20	0
Group G	28dpi	1.00 ± 0.63		10	–	–	–	–
	49dpi	1.00 ± 0.00	7.33 ± 0.58**	10	0**	10**	90**	0
Group H	28dpi	0.00 ± 0.00		0	–	–	–	–
	49dpi	0.00 ± 0.00	0.00 ± 0.00	0	100	100	0	0

**indicates a highly significant difference compared with Group H (*p* < 0.01), *indicates a significant difference compared with Group H (*p* < 0.05), and“-”indicates that no calculation was performed.

#### 3.10.3 Weight gain

The addition of tylvalosin tartrate in the feed (Groups A to F) significantly improved the relative weight gain of infected pigs (*p* < 0.05). Among these, the group administered 62.5 g/1000 kg feed of tylvalosin tartrate enteric-coated granule demonstrated the most favorable relative weight gain effect, reaching 91.9%. This showed no significant difference compared to either the negative control group or the tylvalosin tartrate premix drug control group (*p* > 0.05). This indicates that tylvalosin tartrate enteric-coated granules can alleviate weight gain retardation in diseased pigs, with weight gain at 49 dpi largely restored to levels comparable to the negative control group (Table 13).

**Table 13:** Body weight changes in different groups.

Groups	Body weight prior to administration (kg/head)	Body weight after administration (kg/head)	Average weight gain (kg/head)	Relative weight gain rate (%)
Group A	25.55 ± 1.58	37.35 ± 2.97	11.80 ± 2.39	77.2%
Group B	25.64 ± 1.78	39.26 ± 2.33	13.63 ± 1.30	89.2%
Group C	25.19 ± 1.19	39.43 ± 1.46	14.24 ± 1.28	91.9%
Group D	25.86 ± 2.69	39.60 ± 3.32	13.74 ± 1.03	90.0%
Group E	25.29 ± 1.81	38.76 ± 3.27	13.47 ± 2.07	88.2%
Group F	25.06 ± 1.24	39.11 ± 2.31	14.05 ± 2.10	91.3%
Group G	25.26 ± 1.79	30.87 ± 2.25	5.61 ± 1.71**	36.7%
Group H	30.45 ± 1.71	44.76 ± 1.95	14.31 ± 1.19	100.0%

“*” denotes significant difference compared with the Group H (*p* < 0.05), “**”denotes extremely significant difference compared with the Group H (*p* < 0.01), and no mark indicates non-significant difference compared with the Group H (*p* > 0.05).

## 4 Discussion

This study evaluated the diagnostic and therapeutic strategies against Mhp, highlighting the improved efficacy of a novel enteric-coated tylvalosin tartrate granule. Accurate diagnosis is also critical for effective Mhp management. Our findings confirm that the detection of Mhp is highly dependent on the timing and site of sample collection. We detected Mhp sIgA antibodies in nasal swabs as early as 1 week post-vaccination, however, Mhp antigen was not detectable in upper respiratory tract samples until 28 dpi, coinciding with the peak of clinical signs and lung lesions (Dubosson et al., 2004; Leal Zimmer et al., 2020; Almeida et al., 2021a). This temporal pattern highlights the early role of mucosal immunity and optimal antigen detection.

Further, we evaluated the multiple sample types and found that oropharyngeal swabs yielded the highest Mhp antigen detection rate via qPCR (Silva et al., 2022). This method offers a practical, non-invasive, and reliable alternative to more complex post-mortem lower respiratory tract sampling. Notably, this method remains feasible under routine farm conditions. Further investigation into whether detection rates might vary at earlier or later time points beyond those tested in this study would also be worthwhile. Furthermore, the sIgA-ELISA proved to be an early and sensitive serological assay, capable of distinguishing infection-induced mucosal antibodies from systemic IgG and maternal antibodies, thus providing a valuable diagnostic for early infection stages (Silva et al., 2009; Bai et al., 2018).

Given the reliance on antimicrobials, standardized susceptibility testing is paramount. Adhering to the International Research Programme on Mycoplasma (IRPCM) recommendations (Hannan, 2000), we established that the virulent Mhp strain exhibited greatest susceptibility to macrolides (including tylvalosin), quinolones, and pleuromutilins, which is consistent with other studies (Ter Laak et al., 1991; Vicca et al., 2004; Felde et al., 2018; Zhang et al., 2019; De Jong et al., 2021). Therefore, this study supports their continued use as key clinical options to treat Mhp infections.

A pivotal finding of our *in vitro* analysis was the significant impact of formulation on antibacterial efficacy. The tylvalosin tartrate enteric-coated granules demonstrated a substantially lower MIC (0.015625 μg/mL) compared to the premix formulation (0.125 μg/mL). This attributable may likely due to the enteric coating, which protects the active ingredient from degradation in the stomach, ensuring more effective release and absorption. Moreover, the enteric-coated granules showed higher efficacy in inhibiting the Mhp biofilm formation, a critical virulence factor that facilitates the immune evasion and chronic infection (TASSEW et al., 2017; Jiang et al., 2024). This suggests that formulation advancements can directly augment a drug’s capacity to combat recurrent infections.

The higher in vitro activity of the enteric-coated granules is strongly supported by its pharmacokinetic (PK) properties. Previous multi-dose PK studies in pigs revealed that the high-dose enteric-coated group achieved rapid absorption (Tmax ~1.19 h), a high peak plasma concentration (Cmax ~333.08 ng/mL), and substantial systemic exposure (AUClast ~909.83 h·ng/mL), coupled with slow elimination (T1/2 ~ 4.46 h). The elevated Cmax and AUC are indicative of a greater drug concentration and prolonged retention time at the target site, i.e., lung tissue (Unpublished data; to be published elsewhere). This PK profile ensures that drug levels exceed the MIC for Mhp for an extended duration, providing a robust mechanistic foundation for enhanced pathogen clearance and improved clinical outcomes.

This integrated pharmacokinetics and pharmacodynamics (PK-PD) profile was confirmed in our therapeutic trial. Administration of tylvalosin tartrate enteric-coated granules at 50–100 g/1000 kg feed effectively treated the artificially induced MPS through normalizing the body temperature, reducing pulmonary lesion scores, and significantly ameliorating the slowed weight gain in infected pigs. The therapeutic effect was dose-dependent, however, the lower dose (25 g/1000 kg feed) was inadequate, while the higher dose (100 g/1000 kg feed) did not yield much benefits compared to the intermediate doses. Therefore, we recommend an optimal therapeutic dosage of 50–75 g/1000 kg feed (calculated as tylvalosin), administered continuously for 7 days. This dosage range aligns with the PK data, ensuring the sufficient drug exposure to effectively suppress the Mhp without incurring damaging effects at higher concentrations, and is consistent with the efficacy reported for the premix formulation in a complex Mhp/PRRSV challenge model (Villarreal et al., 2012; Wu et al., 2025).

The present study has certain limitations. This study utilized only one strain of Mhp (Js strain), which may limit its generalizability to field strains with different susceptibility profiles. As established methods for constructing an artificial infection model already exist, we adopted the approach used by the Jiangsu Academy of Agricultural Sciences to construct an artificial infection model and tested the minimum inhibitory concentration of the test compounds against the Js strain. The mechanism of action of the enteric-coated tylvalosin granules warrants further investigation. In the dosage optimization trial, we continued to monitor the subjects for only 2 weeks after discontinuing administration. We are currently compiling further clinical pharmacokinetic data for this investigational drug and are preparing to publish a paper on the subject. In current study trial, primary efficacy endpoints included clinical cure rate, Mhp antigen clearance, and lung lesion scores, supported by secondary indicators such as weight gain and clinical symptom scores. Notably, no significant changes in overt clinical symptoms or in hematological and biochemical parameters were observed in the infected pigs before and after treatment. We hypothesize that the experimental infection induced a sub-clinical, persistent state rather than acute disease, which may indicate the absence of a pronounced systemic inflammatory response. We have to acknowledge that enteric-coated granules (50–75 g/1000 kg of feed, calculated as tylvalosin) and premixes showed identical efficacy in terms of cure rate and antigen clearance, with no significant differences (*p* > 0.05) observed. However, differences were noted in lung lesion scores; the results for Groups C and D were comparable to those of the healthy control group (Group H) (*p* > 0.05), and showed a significant improvement compared to the premix group (Group F) (*p* < 0.05). These results may explain the differences observed in the MIC results between the two different dosage forms. Furthermore, the sample size for anatomical scoring was limited by strict adherence to animal welfare principles, which prioritized minimizing the animal use while achieving the study objectives.

## 5 Conclusion

This study evaluated the anti-Mhp efficacy of a novel enteric-coated granule formulation of tylvalosin tartrate through comprehensive *in vitro* and *in vivo* assessments. The MIC of the 15 antibiotics revealed that tylvalosin, tiamulin, and ciprofloxacin exhibited the strongest antibacterial activity against the Js strain while the tylvalosin tartrate forumlations, the enteric-coated granules exhibited a lower MIC than premix formulation and antibiotics indicating higher bacteriostatic efficacy. Additionally, the enteric-coated granules effectively suppressed the Mhp biofilm formation demonstrating stronger biofilm inhibitory effects. In the dose-screening trial, no significant differences (*p* > 0.05) were observed among groups in weight gain, complete blood count, blood biochemistry, or clinical symptom scores. The therapeutic efficacy of tylvalosin tartrate enteric-coated granules against artificially infected porcine MPS revealed that optimal therapeutic outcomes can be achieved with low-dose antibiotics in practical applications, thereby reducing the drug residues in edible tissues and lowering clinical costs. Based on the findings of this study, we recommend to administer 50 to 75 g of tylvalosin tartrate per 1,000 kg of feed for a continuous 7-days.

## Data Availability

The original contributions presented in the study are included in the article/supplementary material, further inquiries can be directed to the corresponding author/s.
