# Supplementary material for: Enhanced efficacy of tylvalosin tartrate enteric-coated granules against Mycoplasma hyopneumoniae in pigs: in vitro activity and clinical dosage optimization
Source: Front Microbiol. 2026 Jun 2;17:1809157. doi: 10.3389/fmicb.2026.1809157 (PMC13269106; doi:10.3389/fmicb.2026.1809157)
Supplement: Supplementary file 1 [file Supplementary_file_1.doc]

Appendix 1 The Js strain Titer Assay Results
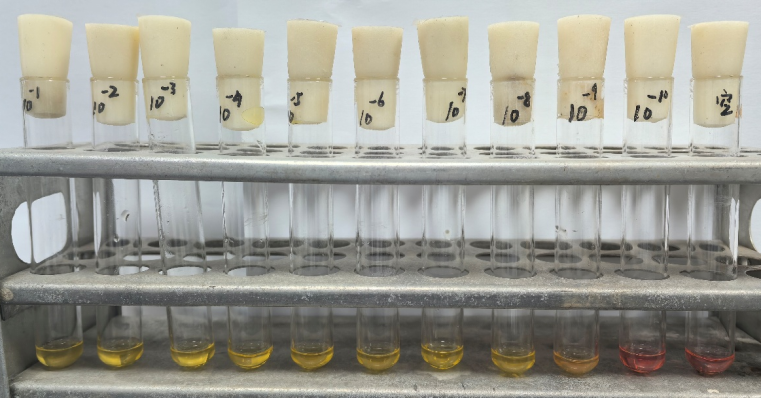


Appendix 2 MIC Determination Results for Mhp Using Two Formulations of Tyvalosin Tartrate


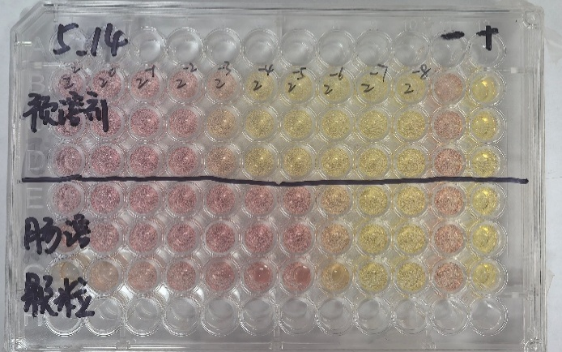


Appendix 3 qPCR-based Mhp Antigen Detection Results

| Well | Well Position | Target Name | CT |
| --- | --- | --- | --- |
| 1 | A1 | P | 24.962 |
| 2 | A2 | S | 33.894 |
| 3 | A3 | S | 33.356 |
| 4 | A4 | S | 31.587 |
| 13 | B1 | N | Undetermined |
| 14 | B2 | S | Undetermined |
| 15 | B3 | S | 28.276 |
| 16 | B4 | S | Undetermined |
| 25 | C1 | S | 20.372 |
| 26 | C2 | S | 34.185 |
| 27 | C3 | S | 5.806 |
| 28 | C4 | S | 23.390 |
| 37 | D1 | S | Undetermined |
| 38 | D2 | S | Undetermined |
| 39 | D3 | S | 31.959 |

Appendix 3 qPCR-based Mhp Antigen Detection Results (Continued)

| Well | Well Position | Target Name | CT |
| --- | --- | --- | --- |
| 40 | D4 | S | 37.423 |
| 49 | E1 | S | 24.607 |
| 50 | E2 | S | 23.756 |
| 51 | E3 | S | 37.159 |
| 52 | E4 | S | Undetermined |
| 61 | F1 | S | 32.539 |
| 62 | F2 | S | 7.402 |
| 63 | F3 | S | 32.140 |
| 64 | F4 | S | 14.054 |
| 73 | G1 | S | 31.134 |
| 74 | G2 | S | Undetermined |
| 75 | G3 | S | 36.603 |
| 76 | G4 | S | Undetermined |
| 85 | H1 | S | Undetermined |
| 86 | H2 | S | Undetermined |
| 87 | H3 | S | 39.508 |
| 88 | H4 | S | Undetermined |

Appendix 4 qPCR-based Mhp Antigen Detection Result

| Well | Well Position | Target Name | CT |
| --- | --- | --- | --- |
| 1 | A1 | S | 30.481 |
| 2 | A2 | S | 32.083 |
| 3 | A3 | S | 30.612 |
| 4 | A4 | S | 21.886 |
| 5 | A5 | S | 34.364 |
| 6 | A6 | S | Undetermined |
| 13 | B1 | P | Undetermined |
| 14 | B2 | S | Undetermined |
| 15 | B3 | S | Undetermined |
| 16 | B4 | S | Undetermined |
| 17 | B5 | S | Undetermined |
| 18 | B6 | S | Undetermined |
| 25 | C1 | N | 6.988 |
| 26 | C2 | S | 34.120 |
| 27 | C3 | S | Undetermined |
| 28 | C4 | S | Undetermined |
| 29 | C5 | S | 37.675 |
| 30 | C6 | S | 10.234 |
| 38 | D2 | S | 29.244 |
| 39 | D3 | S | Undetermined |
| 40 | D4 | S | 26.359 |
| 41 | D5 | S | 6.270 |
| 42 | D6 | S | 9.844 |
| 50 | E2 | S | 26.765 |
| 51 | E3 | S | 33.070 |
| 52 | E4 | S | 33.759 |
| 53 | E5 | S | 34.440 |

Appendix 4 qPCR-based Mhp Antigen Detection Result (Continued)

| Well | Well Position | Target Name | CT |
| --- | --- | --- | --- |
| 54 | E6 | S | Undetermined |
| 62 | F2 | S | 36.315 |
| 63 | F3 | S | 7.378 |
| 64 | F4 | S | Undetermined |
| 65 | F5 | S | 40.795 |
| 66 | F6 | S | 36.442 |
| 74 | G2 | S | 27.433 |
| 75 | G3 | S | 29.951 |
| 76 | G4 | S | 25.970 |
| 77 | G5 | S | 28.636 |
| 78 | G6 | S | 32.029 |
| 86 | H2 | S | 36.794 |
| 87 | H3 | S | 25.817 |
| 88 | H4 | S | 44.763 |
| 89 | H5 | S | Undetermined |
| 90 | H6 | S | Undetermined |
| 91 | H7 | S | Undetermined |

Appendix 5 Gel Electrophoresis Results After PCR Amplification


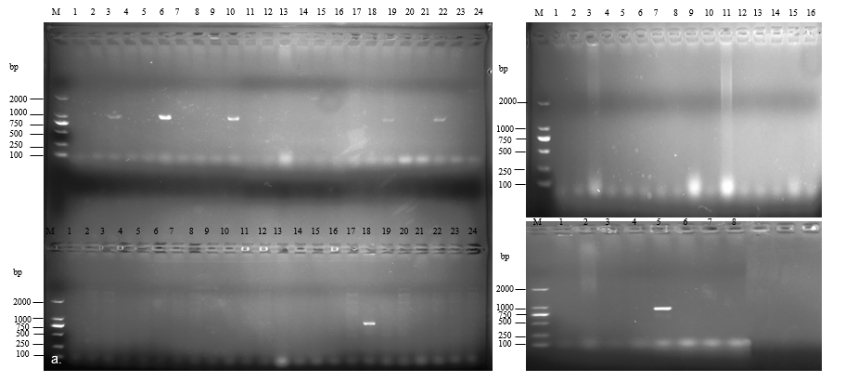


Appendix 6 Typical Clinical Symptoms in Pigs Following Artificial Infection


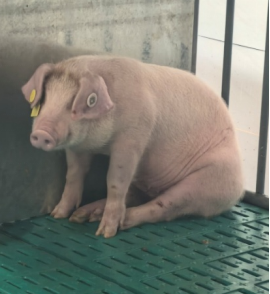


Appendix 7 Mhp Antigen Detection Results Following Artificial Infection

| Well | Well Position | Target Name | CT |
| --- | --- | --- | --- |
| 1 | A1 | P | 17.672 |
| 2 | A2 | S | 29.225 |
| 3 | A3 | S | 35.408 |
| 4 | A4 | S | 18.151 |
| 5 | A5 | S | 26.347 |
| 6 | A6 | S | 33.194 |
| 7 | A7 | S | 18.435 |
| 8 | A8 | S | 18.510 |
| 9 | A9 | S | 20.477 |
| 10 | A10 | S | 21.172 |
| 11 | A11 | S | Undetermined |
| 13 | B1 | P | 15.952 |
| 14 | B2 | S | 31.914 |
| 15 | B3 | S | 27.259 |
| 16 | B4 | S | 22.213 |
| 17 | B5 | S | 21.580 |
| 18 | B6 | S | 22.651 |
| 19 | B7 | S | 22.826 |
| 20 | B8 | S | 16.091 |
| 21 | B9 | S | 20.675 |
| 22 | B10 | S | 24.916 |
| 23 | B11 | S | Undetermined |
| 25 | C1 | N | Undetermined |
| 26 | C2 | S | 28.95271 |
| 27 | C3 | S | 20.747 |
| 28 | C4 | S | 28.086 |
| 29 | C5 | S | 19.638 |

Appendix 7 Mhp Antigen Detection Results Following Artificial Infection (Continued)

| Well | Well Position | Target Name | CT |
| --- | --- | --- | --- |
| 30 | C6 | S | 30.590 |
| 31 | C7 | S | 29.136 |
| 32 | C8 | S | 16.226 |
| 33 | C9 | S | 29.781 |
| 34 | C10 | S | 34.741 |
| 35 | C11 | S | Undetermined |
| 37 | D1 | N | Undetermined |
| 38 | D2 | S | 18.826 |
| 39 | D3 | S | 26.921 |
| 40 | D4 | S | 32.599 |
| 41 | D5 | S | 19.011 |
| 42 | D6 | S | 21.506 |
| 43 | D7 | S | 29.579 |
| 44 | D8 | S | 26.118 |
| 45 | D9 | S | 19.592 |
| 46 | D10 | S | 27.930 |
| 47 | D11 | S | Undetermined |
| 50 | E2 | S | 24.178 |
| 51 | E3 | S | 27.113 |
| 52 | E4 | S | 31.589 |
| 53 | E5 | S | 18.783 |
| 54 | E6 | S | 21.500 |
| 55 | E7 | S | 23.036 |
| 56 | E8 | S | 31.282 |
| 57 | E9 | S | 24.886 |
| 58 | E10 | S | 23.447 |
| 59 | E11 | S | Undetermined |
| 62 | F2 | S | 31.455 |
| 63 | F3 | S | 30.148 |
| 64 | F4 | S | 24.213 |
| 65 | F5 | S | 26.406 |
| 66 | F6 | S | 18.708 |
| 67 | F7 | S | 31.048 |
| 68 | F8 | S | 15.421 |
| 69 | F9 | S | 29.295 |
| 70 | F10 | S | 35.317 |
| 71 | F11 | S | Undetermined |
| 74 | G2 | S | 34.750 |
| 75 | G3 | S | 28.989 |
| 76 | G4 | S | 21.929 |
| 77 | G5 | S | 17.484 |
| 78 | G6 | S | 15.194 |
| 79 | G7 | S | 24.608 |
| 80 | G8 | S | 33.605 |
| 81 | G9 | S | 25.884 |
| 82 | G10 | S | Undetermined |
| 83 | G11 | S | Undetermined |

Appendix 7 Mhp Antigen Detection Results Following Artificial Infection (Continued)

| Well | Well Position | Target Name | CT |
| --- | --- | --- | --- |
| 86 | H2 | S | 33.216 |
| 87 | H3 | S | 20.758 |
| 88 | H4 | S | 28.465 |
| 89 | H5 | S | 35.679 |
| 90 | H6 | S | 28.101 |
| 91 | H7 | S | 25.379 |
| 92 | H8 | S | 38.833 |
| 93 | H9 | S | 21.593 |
| 94 | H10 | S | Undetermined |
| 95 | H11 | S | Undetermined |

Appendix 8 Mhp Antibody Detection Results Following Artificial Infection (96-well plate format)

|  | 1 | 2 | 3 | 4 | 5 | 6 | 7 | 8 | 9 | 10 | 11 |
| --- | --- | --- | --- | --- | --- | --- | --- | --- | --- | --- | --- |
| A | 1.574 | 0.785 | 0.573 | 0.602 | 0.608 | 0.701 | 0.631 | 0.846 | 0.513 | 0.718 | 0.179 |
| B | 1.796 | 0.554 | 0.492 | 0.578 | 0.904 | 1.77 | 0.828 | 1.67 | 0.849 | 0.766 | 0.329 |
| C | 0.173 | 0.842 | 0.89 | 0.803 | 1.59 | 0.829 | 0.717 | 0.817 | 0.758 | 0.826 | 0.312 |
| D | 0.195 | 0.523 | 1.266 | 0.778 | 0.738 | 0.82 | 0.527 | 0.788 | 1.561 | 0.899 | 0.294 |
| E |  | 0.495 | 0.634 | 0.804 | 0.8 | 0.751 | 0.594 | 0.757 | 0.602 | 1.315 | 0.379 |
| F |  | 0.601 | 0.524 | 0.752 | 0.861 | 0.605 | 0.809 | 0.629 | 0.604 | 0.868 | 0.287 |
| G |  | 1.108 | 1.147 | 0.798 | 0.719 | 0.72 | 0.717 | 1.029 | 0.736 | 0.332 | 0.391 |
| H |  | 1.027 | 0.687 | 0.623 | 0.881 | 1.276 | 0.768 | 0.933 | 0.742 | 0.138 | 0.408 |

Appendix 9 Clinical Symptoms in Each Test Group Prior to Drug Administration at 28dpi:

| Group | Number | Body temperature | Respiratory | Status | Total score |
| --- | --- | --- | --- | --- | --- |
| Group A | 1 | 1 | 0 | 0 | 1 |
|  | 2 | 1 | 0 | 0 | 1 |
|  | 3 | 1 | 0 | 0 | 1 |
|  | 4 | 1 | 1 | 1 | 3 |
|  | 5 | 1 | 0 | 0 | 1 |
|  | 6 | 1 | 0 | 0 | 1 |
|  | 7 | 1 | 0 | 0 | 1 |
|  | 8 | 0 | 0 | 0 | 0 |
|  | 9 | 1 | 0 | 0 | 1 |
|  | 10 | 1 | 0 | 0 | 1 |
| Group B | 11 | 1 | 0 | 0 | 1 |
|  | 12 | 1 | 1 | 0 | 2 |
|  | 13 | 1 | 0 | 0 | 1 |
|  | 14 | 1 | 0 | 0 | 1 |
|  | 15 | 1 | 0 | 0 | 1 |
|  | 16 | 1 | 0 | 0 | 1 |
|  | 17 | 1 | 0 | 0 | 1 |
|  | 18 | 0 | 0 | 0 | 0 |
|  | 19 | 1 | 0 | 0 | 1 |
|  | 20 | 1 | 0 | 0 | 1 |
| Group C | 21 | 1 | 0 | 0 | 1 |
|  | 22 | 1 | 1 | 0 | 2 |

Appendix 9 Clinical Symptoms in Each Test Group Prior to Drug Administration at 28dpi: (Continued)

| Group | Number | Body temperature | Respiratory | Status | Total score |
| --- | --- | --- | --- | --- | --- |
| Group C | 23 | 1 | 0 | 0 | 1 |
|  | 24 | 0 | 0 | 0 | 0 |
|  | 25 | 1 | 0 | 0 | 1 |
|  | 26 | 1 | 0 | 0 | 1 |
|  | 27 | 1 | 1 | 0 | 2 |
|  | 28 | 1 | 0 | 0 | 1 |
|  | 29 | 1 | 0 | 0 | 1 |
|  | 30 | 1 | 0 | 0 | 1 |
| Group D | 31 | 1 | 0 | 0 | 1 |
|  | 32 | 1 | 0 | 0 | 1 |
|  | 33 | 1 | 1 | 0 | 2 |
|  | 34 | 1 | 0 | 0 | 1 |
|  | 35 | 0 | 0 | 0 | 0 |
|  | 36 | 1 | 0 | 0 | 1 |
|  | 37 | 1 | 1 | 0 | 2 |
|  | 38 | 1 | 0 | 0 | 1 |
|  | 39 | 1 | 0 | 0 | 1 |
|  | 40 | 1 | 0 | 0 | 1 |
| Group E | 41 | 1 | 0 | 0 | 1 |
|  | 42 | 1 | 0 | 0 | 1 |
|  | 43 | 0 | 0 | 0 | 0 |
|  | 44 | 1 | 0 | 0 | 1 |
|  | 45 | 1 | 1 | 0 | 2 |
|  | 46 | 1 | 0 | 0 | 1 |
|  | 47 | 1 | 0 | 0 | 1 |
|  | 48 | 0 | 0 | 0 | 0 |
|  | 49 | 1 | 0 | 0 | 1 |
|  | 50 | 1 | 0 | 0 | 1 |
| Group F | 51 | 1 | 0 | 0 | 1 |
|  | 52 | 1 | 0 | 0 | 1 |
|  | 53 | 0 | 0 | 0 | 0 |
|  | 54 | 1 | 0 | 0 | 1 |
|  | 55 | 1 | 1 | 0 | 2 |
|  | 56 | 0 | 0 | 0 | 0 |
|  | 57 | 1 | 0 | 0 | 1 |
|  | 58 | 1 | 0 | 0 | 1 |
|  | 59 | 1 | 0 | 0 | 1 |
|  | 60 | 1 | 0 | 0 | 1 |
| Group G | 61 | 1 | 0 | 0 | 1 |
|  | 62 | 1 | 0 | 0 | 1 |
|  | 63 | 1 | 1 | 0 | 2 |
|  | 64 | 1 | 0 | 0 | 1 |
|  | 65 | 0 | 0 | 0 | 0 |
|  | 66 | 1 | 0 | 0 | 1 |
|  | 67 | 1 | 1 | 0 | 2 |
|  | 68 | 0 | 0 | 0 | 0 |

Appendix 9 Clinical Symptoms in Each Test Group Prior to Drug Administration at 28dpi: (Continued)

| Group | Number | Body temperature | Respiratory | Status | Total score |
| --- | --- | --- | --- | --- | --- |
| Group G | 69 | 1 | 0 | 0 | 1 |
|  | 70 | 1 | 0 | 0 | 1 |
| Group H | 71 | 0 | 0 | 0 | 0 |
|  | 72 | 0 | 0 | 0 | 0 |
|  | 73 | 0 | 0 | 0 | 0 |
|  | 74 | 0 | 0 | 0 | 0 |
|  | 75 | 0 | 0 | 0 | 0 |
|  | 76 | 0 | 0 | 0 | 0 |
|  | 77 | 0 | 0 | 0 | 0 |
|  | 78 | 0 | 0 | 0 | 0 |
|  | 79 | 0 | 0 | 0 | 0 |
|  | 80 | 0 | 0 | 0 | 0 |

Appendix 10 Clinical Symptoms in Each Treatment Group at 49 dpi:

| Group | Number | Body temperature | Respiratory | Status | Total score |
| --- | --- | --- | --- | --- | --- |
| Group A | 1 | 1 | 0 | 0 | 1 |
|  | 2 | 0 | 0 | 0 | 0 |
|  | 3 | 0 | 0 | 0 | 0 |
|  | 4 | 0 | 0 | 0 | 0 |
|  | 5 | 1 | 0 | 0 | 1 |
|  | 6 | 1 | 0 | 0 | 1 |
|  | 7 | 0 | 0 | 0 | 0 |
|  | 8 | 0 | 0 | 0 | 0 |
|  | 9 | 1 | 0 | 0 | 1 |
|  | 10 | 1 | 0 | 0 | 1 |
| Group B | 11 | 1 | 0 | 0 | 1 |
|  | 12 | 0 | 0 | 0 | 0 |
|  | 13 | 1 | 0 | 0 | 1 |
|  | 14 | 0 | 0 | 0 | 0 |
|  | 15 | 0 | 0 | 0 | 0 |
|  | 16 | 1 | 0 | 0 | 1 |
|  | 17 | 1 | 0 | 0 | 1 |
|  | 18 | 0 | 0 | 0 | 0 |
|  | 19 | 0 | 0 | 0 | 0 |
|  | 20 | 0 | 0 | 0 | 0 |
| Group C | 21 | 0 | 0 | 0 | 0 |
|  | 22 | 1 | 0 | 0 | 1 |
|  | 23 | 0 | 0 | 0 | 0 |
|  | 24 | 0 | 0 | 0 | 0 |
|  | 25 | 0 | 0 | 0 | 0 |
|  | 26 | 0 | 0 | 0 | 0 |
|  | 27 | 1 | 0 | 0 | 1 |
|  | 28 | 0 | 0 | 0 | 0 |
|  | 29 | 0 | 0 | 0 | 0 |
|  | 30 | 0 | 0 | 0 | 0 |
| Group D | 31 | 0 | 0 | 0 | 0 |

Appendix 10 Clinical Symptoms in Each Treatment Group at 49 dpi: (Continued)

| Group | | Number | | Body temperature | | Respiratory | | Status | | Total score |
| --- | --- | --- | --- | --- | --- | --- | --- | --- | --- | --- |
| Group D | 32 | | 0 | | 0 | | 0 | | 0 | |
|  | 33 | | 0 | | 0 | | 0 | | 0 | |
|  | 34 | | 0 | | 0 | | 0 | | 0 | |
|  | 35 | | 1 | | 0 | | 0 | | 1 | |
|  | 36 | | 0 | | 0 | | 0 | | 0 | |
|  | 37 | | 1 | | 0 | | 0 | | 1 | |
|  | 38 | | 0 | | 0 | | 0 | | 0 | |
|  | 39 | | 0 | | 0 | | 0 | | 0 | |
|  | 40 | | 0 | | 0 | | 0 | | 0 | |
| Group E | 41 | | 0 | | 0 | | 0 | | 0 | |
|  | 42 | | 0 | | 0 | | 0 | | 0 | |
|  | 43 | | 1 | | 0 | | 0 | | 1 | |
|  | 44 | | 0 | | 0 | | 0 | | 0 | |
|  | 45 | | 0 | | 0 | | 0 | | 0 | |
|  | 46 | | 1 | | 0 | | 0 | | 1 | |
|  | 47 | | 1 | | 0 | | 0 | | 1 | |
|  | 48 | | 0 | | 0 | | 0 | | 0 | |
|  | 49 | | 0 | | 0 | | 0 | | 0 | |
|  | 50 | | 0 | | 0 | | 0 | | 0 | |
| Group F | 51 | | 0 | | 0 | | 0 | | 0 | |
|  | 52 | | 0 | | 0 | | 0 | | 0 | |
|  | 53 | | 1 | | 0 | | 0 | | 1 | |
|  | 54 | | 0 | | 0 | | 0 | | 0 | |
|  | 55 | | 0 | | 0 | | 0 | | 0 | |
|  | 56 | | 0 | | 0 | | 0 | | 0 | |
|  | 57 | | 1 | | 0 | | 0 | | 1 | |
|  | 58 | | 0 | | 0 | | 0 | | 0 | |
|  | 59 | | 0 | | 0 | | 0 | | 0 | |
|  | 60 | | 0 | | 0 | | 0 | | 0 | |
| Group G | 61 | | 1 | | 0 | | 0 | | 1 | |
|  | 62 | | 1 | | 0 | | 0 | | 1 | |
|  | 63 | | 1 | | 0 | | 0 | | 1 | |
|  | 64 | | 1 | | 0 | | 0 | | 1 | |
|  | 65 | | 1 | | 0 | | 0 | | 1 | |
|  | 66 | | 1 | | 0 | | 0 | | 1 | |
|  | 67 | | 1 | | 0 | | 0 | | 1 | |
| Group G | 68 | | 1 | | 0 | | 0 | | 1 | |
|  | 69 | | 1 | | 0 | | 0 | | 1 | |
|  | 70 | | 1 | | 0 | | 0 | | 1 | |
| Group H | 71 | | 0 | | 0 | | 0 | | 0 | |
|  | 72 | | 0 | | 0 | | 0 | | 0 | |
|  | 73 | | 0 | | 0 | | 0 | | 0 | |
|  | 74 | | 0 | | 0 | | 0 | | 0 | |
|  | 75 | | 0 | | 0 | | 0 | | 0 | |
|  | 76 | | 0 | | 0 | | 0 | | 0 | |

Appendix 10 Clinical Symptoms in Each Treatment Group at 49 dpi: (Continued)

| Group | | Number | | Body temperature | | Respiratory | | Status | | Total score |
| --- | --- | --- | --- | --- | --- | --- | --- | --- | --- | --- |
| Group H | 77 | | 0 | | 0 | | 0 | | 0 | |
|  | 78 | | 0 | | 0 | | 0 | | 0 | |
|  | 79 | | 0 | | 0 | | 0 | | 0 | |
|  | 80 | | 0 | | 0 | | 0 | | 0 | |

Appendix 11 Mhp Antigen Test Results at 49 dpi:

| Well | Well Position | Target Name | CT |
| --- | --- | --- | --- |
| 1 | A1 | P | 23.265 |
| 2 | A2 | S | 23.056 |
| 3 | A3 | S | 21.110 |
| 4 | A4 | S | 26.211 |
| 5 | A5 | S | Undetermined |
| 6 | A6 | S | Undetermined |
| 7 | A7 | S | Undetermined |
| 8 | A8 | S | Undetermined |
| 9 | A9 | S | 24.057 |
| 10 | A10 | S | 29.539 |
| 11 | A11 | S | Undetermined |
| 13 | B1 | P | 22.324 |
| 14 | B2 | S | Undetermined |
| 15 | B3 | S | 21.065 |
| 16 | B4 | S | 49.195 |
| 17 | B5 | S | Undetermined |
| 18 | B6 | S | Undetermined |
| 19 | B7 | S | Undetermined |
| 20 | B8 | S | Undetermined |
| 21 | B9 | S | 63.319 |
| 22 | B10 | S | 20.220 |
| 23 | B11 | S | Undetermined |
| 25 | C1 | N | Undetermined |
| 26 | C2 | S | Undetermined |
| 27 | C3 | S | 31.270 |
| 28 | C4 | S | Undetermined |
| 29 | C5 | S | 24.754 |
| 30 | C6 | S | 17.739 |
| 31 | C7 | S | 22.006 |
| 32 | C8 | S | Undetermined |
| 33 | C9 | S | Undetermined |
| 34 | C10 | S | 27.183 |
| 35 | C11 | S | Undetermined |
| 37 | D1 | N | Undetermined |
| 38 | D2 | S | Undetermined |
| 39 | D3 | S | Undetermined |
| 40 | D4 | S | Undetermined |
| 41 | D5 | S | Undetermined |
| 42 | D6 | S | Undetermined |

Appendix 11 Mhp Antigen Test Results at 49 dpi: (Continued)

| Well | Well Position | Target Name | CT |
| --- | --- | --- | --- |
| 43 | D7 | S | Undetermined |
| 44 | D8 | S | Undetermined |
| 45 | D9 | S | Undetermined |
| 46 | D10 | S | 22.150 |
| 47 | D11 | S | 57.931 |
| 50 | E2 | S | 27.297 |
| 51 | E3 | S | 23.313 |
| 52 | E4 | S | Undetermined |
| 53 | E5 | S | Undetermined |
| 54 | E6 | S | 35.407 |
| 55 | E7 | S | Undetermined |
| 56 | E8 | S | Undetermined |
| 57 | E9 | S | 31.314 |
| 58 | E10 | S | 34.300 |
| 59 | E11 | S | Undetermined |
| 62 | F2 | S | 28.673 |
| 63 | F3 | S | Undetermined |
| 64 | F4 | S | 21.591 |
| 65 | F5 | S | Undetermined |
| 66 | F6 | S | Undetermined |
| 67 | F7 | S | 30.206 |
| 68 | F8 | S | 63.520 |
| 69 | F9 | S | 34.888 |
| 70 | F10 | S | 27.631 |
| 71 | F11 | S | Undetermined |
| 74 | G2 | S | Undetermined |
| 75 | G3 | S | Undetermined |
| 76 | G4 | S | Undetermined |
| 77 | G5 | S | Undetermined |
| 78 | G6 | S | Undetermined |
| 79 | G7 | S | 18.991 |
| 80 | G8 | S | Undetermined |
| 81 | G9 | S | 24.855 |
| 82 | G10 | S | Undetermined |
| 83 | G11 | S | Undetermined |
| 86 | H2 | S | Undetermined |
| 87 | H3 | S | 26.902 |
| 88 | H4 | S | Undetermined |
| 89 | H5 | S | Undetermined |
| 90 | H6 | S | Undetermined |
| 91 | H7 | S | Undetermined |
| 92 | H8 | S | Undetermined |
| 93 | H9 | S | 29.936 |
| 94 | H10 | S | Undetermined |
| 95 | H11 | S | Undetermined |
